# Distinguishing shadows from surface boundaries using local achromatic cues

**DOI:** 10.1371/journal.pcbi.1010473

**Published:** 2022-09-14

**Authors:** Christopher DiMattina, Josiah J. Burnham, Betul N. Guner, Haley B. Yerxa

**Affiliations:** 1 Computational Perception Laboratory, FGCU Computational Facility, & Department of Psychology, Florida Gulf Coast University, Fort Myers, Florida, United States of America; 2 Computational Perception Laboratory & Department of Software Engineering, Florida Gulf Coast University, Fort Myers, Florida, United States of America; 3 Computational Perception Laboratory & Department of Psychology, Florida Gulf Coast University, Fort Myers, Florida, United States of America; Cornell University, UNITED STATES

## Abstract

In order to accurately parse the visual scene into distinct surfaces, it is essential to determine whether a local luminance edge is caused by a boundary between two surfaces or a shadow cast across a single surface. Previous studies have demonstrated that local chromatic cues may help to distinguish edges caused by shadows from those caused by surface boundaries, but the information potentially available in local *achromatic* cues like contrast, texture, and penumbral blur remains poorly understood. In this study, we develop and analyze a large database of hand-labeled achromatic shadow edges to better understand what image properties distinguish them from occlusion edges. We find that both the highest contrast as well as the lowest contrast edges are more likely to be occlusions than shadows, extending previous observations based on a more limited image set. We also find that contrast cues alone can reliably distinguish the two edge categories with nearly 70% accuracy at 40x40 resolution. Logistic regression on a Gabor Filter bank (**GFB**) modeling a population of V1 simple cells separates the categories with nearly 80% accuracy, and furthermore exhibits tuning to penumbral blur. A Filter-Rectify Filter (**FRF**) style neural network extending the **GFB** model performed at better than 80% accuracy, and exhibited blur tuning and greater sensitivity to texture differences. We compare human performance on our edge classification task to that of the **FRF** and **GFB** models, finding the best human observers attaining the same performance as the machine classifiers. Several analyses demonstrate both classifiers exhibit significant positive correlation with human behavior, although we find a slightly better agreement on an image-by-image basis between human performance and the **FRF** model than the **GFB** model, suggesting an important role for texture.

## Author summary

Distinguishing edges caused by changes in illumination from edges caused by surface boundaries is an essential computation for accurately parsing the visual scene. Previous psychophysical investigations examining the utility of various locally available cues to classify edges as shadows or surface boundaries have primarily focused on color, as surface boundaries often give rise to more significant change in color than shadows. However, even in grayscale images we can readily distinguish shadows from surface boundaries, suggesting an important role for achromatic cues in addition to color. We demonstrate using statistical analysis of natural shadow and surface boundary edges that locally available achromatic cues can be exploited by machine classifiers to reliably distinguish these two edge categories. These classifiers exhibit sensitivity to blur and local texture differences, and exhibit reasonably good agreement with humans classifying edges as shadows or surface boundaries. As trichromatic vision is relatively rare in the animal kingdom, our work suggests how organisms lacking rich color vision can still exploit other cues to avoid mistaking illumination changes for surface changes.

## Introduction

Luminance edge detection is an essential visual computation for identifying the boundary between two surfaces [1–4]. However, it is well known that in natural images, luminance edges may arise from several causes other than surface boundaries: Specular reflections, changes in material properties, changes in surface orientation, and cast shadows all can give rise to changes in luminance that do not correspond to surface boundaries [5–8]. Shadows are one class of luminance edge which are essential to distinguish from other potential causes, since shadows do not indicate a change in surface reflectance properties, but simply a change in illumination [9–11].

Recent work has addressed the question of how the visual system distinguishes material or reflectance edges from shadow edges in natural vision [12]. In this study, human observers classified both color and grayscale image patches viewed through an aperture as either shadow edges or material edges. These authors found that observers performed significantly better with the color images, and that a machine classifier making use of chromatic cues outperformed a machine classifier only using luminance cues. These findings are consistent with several studies suggesting an important role for color in distinguishing illumination from material [10,11]. In addition to improved performance with chromatic information, these investigators also observed improved performance with increasing aperture size. This may be because at larger spatial scales, texture information becomes available which is not available at smaller spatial scales.

**Fig 1:**
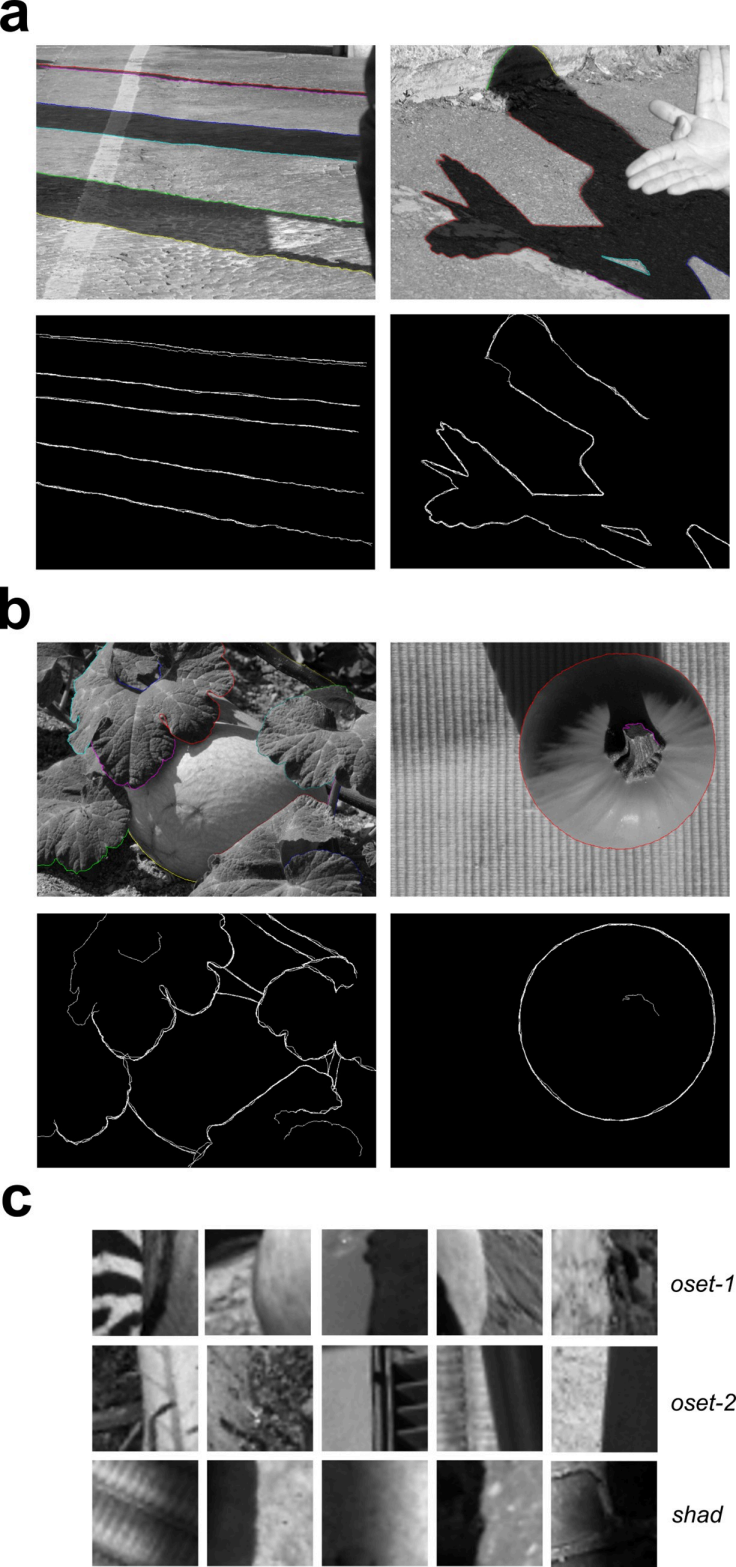
Images and 40x40 patches from the shadow and occlusion databases. (**a**) *Top*: Two representative images from our shadow database (**shad**) with shadow edges labeled by one observer. *Bottom*: Overlaid shadow boundaries obtained from four observers. (**b**) *Top*: Two representative images from **oset-2**, with occlusions labeled by one observer. *Bottom*: Overlaid occlusion boundaries from three observers. (**c**) Representative 40x40 image patches from each image set.

Here we address the question of what locally available *achromatic* information is available to distinguish surface occlusion boundaries from cast shadows. This question is important since distinguishing material properties from the illuminant is a universal problem that all visual systems face, yet trichromacy is relatively rare in the animal kingdom. Most mammals are dichromats, and some mammals (for instance, owl monkeys) are cone monochromats. For such organisms, chromatic cues will be less useful, or even completely unavailable, for shadow segmentation. Furthermore, even in trichromatic species, color information is by no means necessary to segment shadows, as a casual inspection of the grayscale images in **Fig 1A** reveals. This question is also important for the development of computer vision methods for shadow segmentation and removal in grayscale images. Most algorithms developed for this purpose utilize color information to distinguish changes in illumination from changes in surface reflectance (e.g., [9]). However, in some computer vision applications chromatic information may be unavailable, for instance when processing archival images which pre-date color photography.

At least three possible sources of information are potentially available: (1) Differences in luminance contrast, (2) Differences in spatial luminance profiles, and (3) Texture cues. Pursuant to (1), quantitative Bayesian classifier analyses demonstrate that on average occlusions can be distinguished from non-occlusions (with shadows being one kind of non-occlusion edge) using only Michaelson contrast [7]. However, in this study a fairly limited set of images was used, and it did not focus specifically on shadows. Pursuant to (2), it is well known that the luminance edges arising from shadows are often more gradual than those arising from surface boundaries due to penumbral blur [7,8], and furthermore human observers can reliably distinguish sharp and blurred edges [13]. Pursuant to (3), although texture segmentation is extremely well studied both psychophysically and computationally [14,15], to our knowledge no psychophysical work has directly considered the role of local texture cues for classifying shadow and occlusion edges.

To address these questions, we develop a publicly available database of shadow edges by hand-labeling a set of 47 natural images from the McGill Color Calibrated Image Database [9]. We compare basic contrast and spatial frequency statistics of these shadow edges to the same properties measured from a novel occlusion database derived from a subset of 20 of the 47 images, as well as a previously published database of surface boundaries (mostly depth occlusions) containing 100 images [3]. We demonstrate that machine classifiers defined using only 2 or 3 statistics can reliably discriminate 40x40 patches containing shadows from patches containing surface boundaries at nearly 70% accuracy when generalizing to images not used for training. We then evaluated the ability of an image-computable model comprised of a population of multi-scale Gabor filters resembling V1 simple cells to distinguish these two categories, attaining nearly 80% accuracy when generalizing to images not used for training. Furthermore, we found that our Gabor filter bank (**GFB**) model exhibited clear tuning to penumbral blur, a cue which often accompanies shadows. To test the hypothesis that local texture cues may be used to distinguish shadows from surface boundaries, we next trained a Filter-Rectify-Filter (**FRF**) style neural network model [14,16–20] to solve the classification problem. Our best **FRF** model could classify 40x40 image patches taken from novel images at better than 80% accuracy on average. Analysis of the hidden units in these trained models demonstrated that many represented spatial differences in texture. Additional testing of the **GFB** and **FRF** models revealed reduced data-fitting performance with the removal of texture information, further underscoring the importance of local textures cues.

We performed a simple psychophysical experiment where observers classified 40x40 grayscale image patches as surface boundaries or shadow edges. We found that the best human observers performed about as well as the machine classifiers (**GFB**, **FRF**) performed generalizing to novel images (80% accuracy). By correlating human and model classification performance on an image-by-image basis, we observe strong positive correlations between each classifier and human behavior. However, we observed a stronger correlation between human behavior and the **FRF** model predictions than the **GFB** model, even though the generalization performance of the **FRF** model (as measured by percent correct) was only slightly better than the **GFB** model. Both the **FRF** model and the **GFB** models are sensitive to penumbral blur, but the **FRF** model exhibits a stronger sensitivity to texture cues. Therefore, these results suggest that human observers are making use of textural cues to classify luminance changes as shadows or surface boundaries.

Overall, our results show that there are multiple, locally available achromatic cues which the visual system can potentially exploit to distinguish luminance changes arising from shadows from those caused by surface boundaries. It is of great interest for future work to better understand how human observers exploit this information for this crucial natural vision task, and we propose a research strategy for future psychophysical experiments.

## Results

### Shadow edge database

Four observers hand-labeled all of the shadow boundaries in a set of 47 images (786 x 576 pixel resolution) taken from the McGill calibrated color image database [9]. Two images from our shadow database (**shad**), together with the shadow edges annotated by one observer, are shown in the top panels of **Fig 1A.** A binary overlay of the labels obtained from all four observers is shown in the bottom panels of **Fig 1A.** To compare the statistical properties of shadow edges with surface boundaries, we utilized a previously developed database [3] of hand-labeled surface boundaries taken from 100 images in the McGill Dataset (**oset-1**). The **oset-1** database is primarily comprised of true occlusion boundaries, in which one surface blocks or occludes another surface which is displaced in depth. However, many of the boundaries in this database not actually depth occlusions, but rather boundaries between two image regions juxtaposed in the same depth plane, for instance, a patch of grass next to a patch of dirt (**S1 Fig**). Such boundaries occurring in the absence of a depth discontinuity might be more accurately described as more general “material changes” [12]. Nevertheless, in the present paper, we will heretofore take the term “occlusion” to broadly encompasses surface boundaries that are either depth occlusions (the overwhelming majority of our database) or surface juxtapositions.

In addition to **oset-1**, we also developed a second occlusion database (**oset-2**) from a subset of shadow database images (20 out of 47 images) by having the three of the four observers who labeled the shadow also hand-label occlusions in these images. Two images from **oset-2** along with their hand-labeled occlusions are shown in **Fig 1B**, and examples of image patches randomly selected from each dataset can be seen in **Fig 1C**. The total number of hand-labeled occlusions and shadows in these images is quite large, with the **shad** database containing a total of 272,043 labeled shadow patches in 47 images. **oset-1** contains over 1.25 million occlusion patches (1,258,000), and **oset-2** contains 84,112 occlusion patches. However, since a “patch” is defined by the location of its center pixel, two patches centered on adjacent pixels will exhibit substantial overlap. All databases (**shad**, **oset-1**, **oset-2**) are freely available at: https://www.fgcu.edu/faculty/cdimattina/.

### Analysis of image patch statistics

#### Contrast statistics

Previous investigators have characterized some of the statistical properties of occlusion edges and non-occlusion edges (including shadows) in natural scenes [7,12]. To connect with these previous explorations, we measured some of these quantities from both the occlusion and shadow edges: (1) RMS contrast *c*_*RMS*_ (**Eq 2**), and (2) Michelson contrast *c*_*M*_ (**Eq 3**). All analyses were performed on 40x40 pixel (5% of larger image dimension) image patches from one of our databases (**shad**, **oset-1**, **oset-2**), with the constraint that all patches were centered on a human-labeled edge (shadow or occlusion).

**Fig 2:**
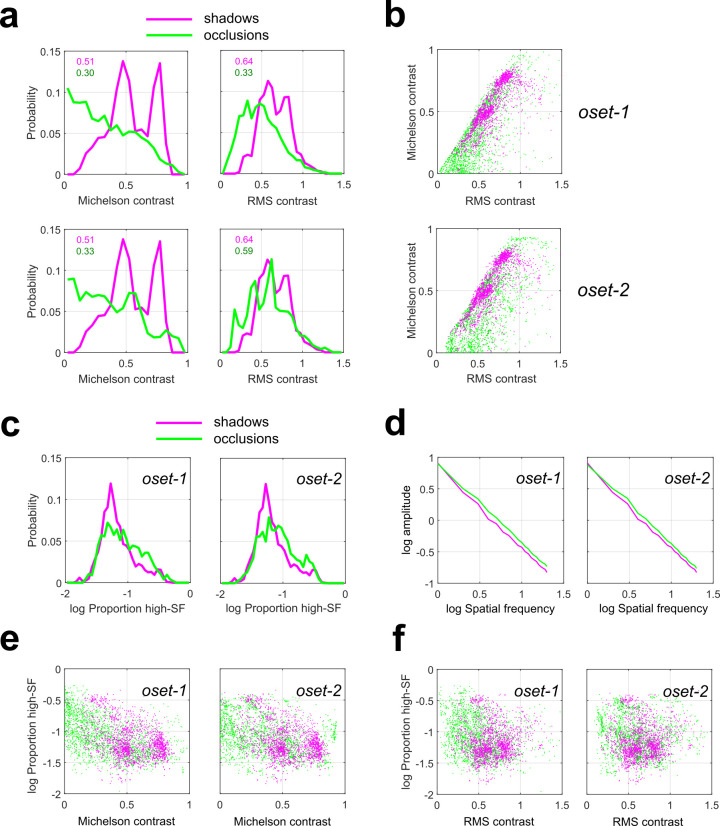
Contrast statistics measured from shadow and occlusion edges (N = 2000 of each category). Green curves/symbols indicate occlusions, Magenta curves/symbols indicate shadows. (**a**) Distributions of Michelson contrast (*left*) and RMS contrast (*right*) for our shadow database and two sets of occlusions (**oset-1**: *top*, **oset-2**: *bottom*). Note that for both occlusion sets, the highest and lowest contrast edges are occlusions. (**b**) Two-dimensional scatterplot of contrast measurements for shadow and occlusion edges for both sets of occlusions. (**c**) Probability distribution of stimulus power *π*_*h*_ in the high spatial frequency (>10 cycles/image) range for our shadow database (magenta curves) and both occlusion sets (green curves). (**d**) Rotational average of the amplitude spectrum for shadows (magenta curves) and both occlusion sets (green curves). (**e**) Scatterplots of *π*_*h*_ and Michelson contrast *c*_*M*_ for shadows (magenta symbols) and both occlusion sets (green symbols). (**f**) Same as (c) but for RMS contrast *c*_*RMS*_.

**Fig 2A** plots the overlaid probability distributions of these quantities for the shadow images (magenta curves) and both occlusion databases (**oset-1**: *top*, **oset-2**: *bottom*, green curves). We see that the distribution of Michelson contrast (*c*_*M*_) for occlusions (green lines) spans the entire range of observed values, with occlusion edges giving rise to both the highest and lowest values. Furthermore, we see that occlusion edges are much more likely than shadow edges (magenta lines) to have very low values of *c*_*M*_. We obtained median values of *c*_*M*_ = 0.51 for our shadow set, and *c*_*M*_ = 0.30, 0.33 for **oset-1** and **oset-2**, respectively. Median values are significantly different between shadows and both occlusion sets (rank-sum test, *p* < 0.001). Similar results were obtained for RMS contrast (*c*_*RMS*_), although in this case the discrepancy between categories was smaller, and the distribution for occlusions was closer in shape to a normal distribution. For RMS contrast, we obtained median values of *c*_*RMS*_ = 0.64 for the shadows and *c*_*RMS*_ = 0.47, 0.59 respectively. Median values are significantly different between shadows and both occlusion sets (rank-sum test, *p* < 0.001). Large and highly significant positive correlations (Pearson’s *r*) were observed between these two contrast measures for all datasets (**oset-1**: *r* = 0.754, *p* < 0.001; **oset-2**: *r* = 0.722, *p* < 0.001, **shad**: *r* = 0.743, *p* < 0.001).

Our finding that the lowest-contrast edges (by both measures) were occlusions rather than shadows is consistent with the fact that occlusion edges can often arise from two surfaces having identical mean luminance [3], whereas by definition a shadow edge requires a change in luminance. However, in agreement with previous measurements using a different set of occlusion patches identified using different methodology [7], we see that the far-right tails of the contrast distributions are dominated by occlusions, meaning that the highest contrast edges are more likely to be occlusions rather than shadows. Since 19 of the 38 images analyzed in [7] were also contained in **oset-1**, we repeated our analysis on this subset of images, finding results consistent with our analyses based on the full image set (**S2 Fig**).

We also see from **Fig 2A** that in our sample the distribution of *c*_*M*_ (but not *c*_*RMS*_) appears to have some degree of bimodality. Most likely this reflects the properties of our database rather than the physics of shadow formation, as our shadow set contained a sub-set of images taken in conditions of strong illumination from a point source (the sun). Comparing the green curves on the top and bottom of **Fig 2A**, we see a strong qualitative consistency between both sets of occlusion images. This provides us with reassurance that our measurements of contrast statistics reflect the general properties of occlusion edges rather than the idiosyncrasies of our databases.

**Fig 2B** shows scatterplots of our measured quantities (*c*_*M*_, *c*_*RMS*_) for both occlusion (green symbols) and shadow edges (magenta) for both sets of occlusions. For **oset-1**, a classifier using these two cues can separate occlusions and shadows with an average performance of 67.7% accuracy on test image sets not used for classifier training (taken from different images, see **METHODS**), and 69.9% accuracy on the training sets (**S1 Table**). For **oset-2**, the classifier separates the two categories in the test sets with an average accuracy of 68.5%, with average accuracy of 70.7% on the training sets (**S2 Table**). Similar results were obtained when the training and test sets were both sampled uniformly (with replacement) from all images (see **METHODS**). In this analysis, the classifier trained on 16,000 image patches predicts test set category (4000 patches) with accuracy of 68.7% for **oset-1**, and 69.6% for **oset-2**.

**Fig 3:**
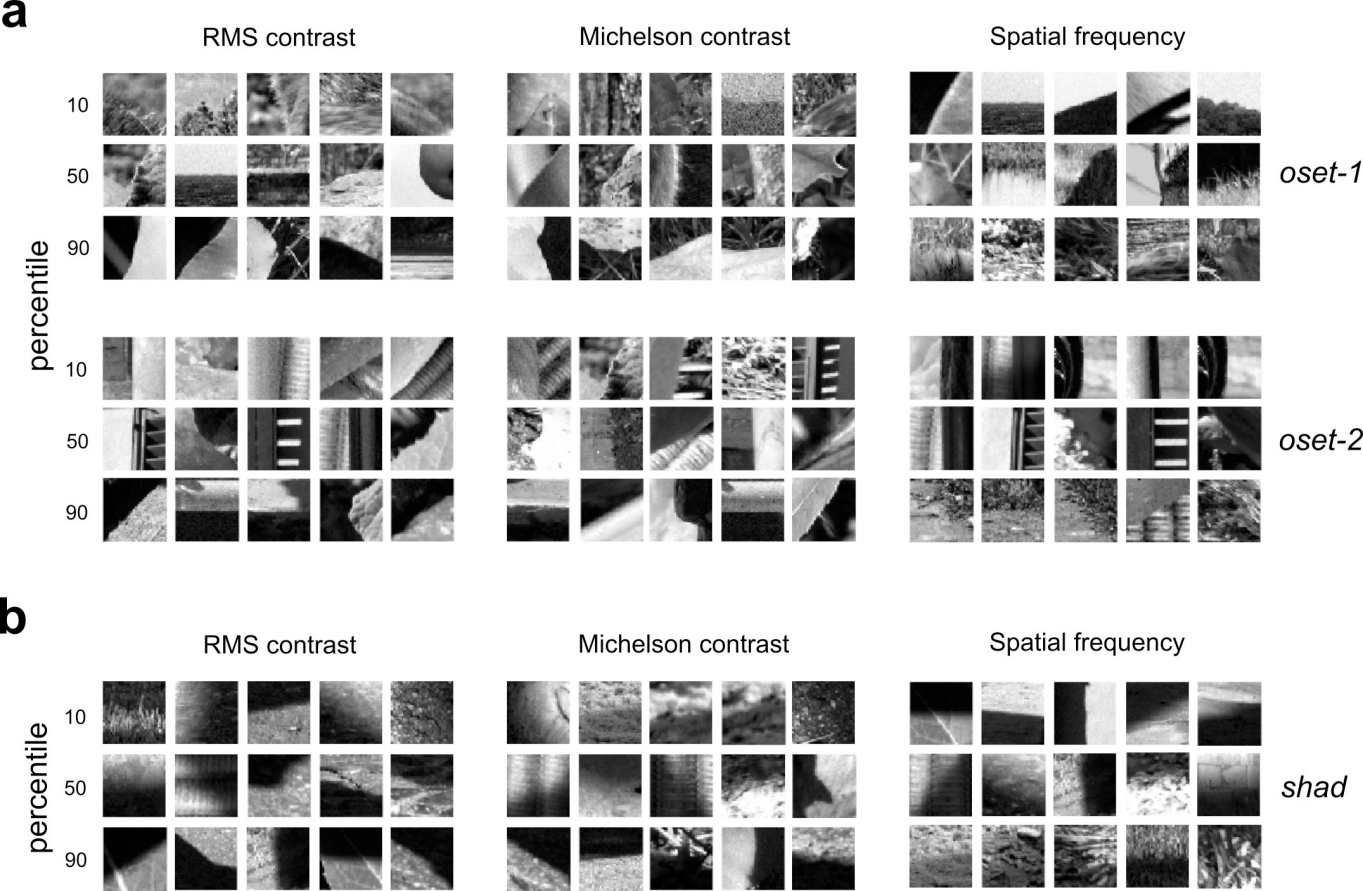
Example occlusion and shadow edges at varying percentiles of our contrast and spatial frequency measurements. Each image patch is within 8 ranks of the indicated percentile (out of N = 2000 total image patches analyzed). (**a**) Occlusion edges at varying percentiles of each contrast measure (RMS: *left*; Michelson: *center*) and our spatial frequency measure (*π*_*h*_: *right*) for both sets of occlusions (**oset-1**: *top*, **oset-2**: *bottom*). (**b**) Same as (a), but for shadow edges from our database.

**Fig 3** (*left + center*) illustrates examples of image patches near the 10^th^, 50^th^, and 90^th^ percentiles when the images are sorted by each contrast measure (each patch is at most 8 ranks from the exact percentile rank). **Fig 3A** shows occlusion images from both sets, and **Fig 3B** shows the shadow images. In our analyses, we applied our measurements to the grayscale converted (**Eq 1**) RGB values obtained from the linearized images without any pre-normalization. This is because such operations may potentially reduce contrast cues. However, repeating these analyses where each individual linearized image patch was also normalized to lie in the range [0, 1] yielded nearly identical results (**S3 Fig**).

#### Spatial frequency statistics

As we see from **Figs 1C** and **3**, another potential cue for distinguishing shadow edges from occlusions is that many shadows exhibit a gradual transition between luminance levels due to penumbral blur [8]. Therefore, we should expect that edges arising from shadows may have less energy in higher spatial frequency (SF) ranges than those arising from occlusions. Analysis of the proportion *π*_*h*_ of stimulus energy at higher spatial frequencies (which we define as those frequencies above 10 cycles/image, with 20 cycles/image being the Nyquist limit) reveals that indeed there are significant differences between occlusions and shadows (**Fig 2C**). Comparison of *π*_*h*_ distribution medians (rank-sum test) yielded significant results for both **oset-1** (occlusion median = 0.076, shadow median = 0.060, *p* < 0.001) and **oset-2** (occlusion median = 0.078, shadow median = 0.060, *p* < 0.001). Averaging the amplitude spectrum obtained from each patch (**Fig 2D**) reveals more energy at higher spatial frequencies for the occlusion images compared to shadow images. **Fig 2E and 2F** shows scatterplots of *π*_*h*_ versus *c*_*M*_ and *c*_*RMS*_ for both **oset-1** (*left*) **and oset-2** (*right*). Significant negative correlations were observed between both contrast measures and *π*_*h*_ for both occlusion sets and the shadow set. That is, higher contrast edges had a smaller proportion of their energy in high spatial frequencies. For occlusion **oset-1**, we obtain correlations between *π*_*h*_ and *c*_*M*_, *c*_*RMS*_ of -0.492, -0.261, respectively (*p* < 0.001 both tests). For occlusion **oset-2**, we a obtain correlations between *π*_*h*_ and *c*_*M*_, *c*_*RMS*_ of -0.315, -0.145, respectively (*p* < 0.001 both tests). For the **shad** set, we obtain correlations between *π*_*h*_ and *c*_*M*_, *c*_*RMS*_ of -0.414 (*p* < 0.001) and -0.052 (*p* = 0.002).

**Fig 3A** (*right*) shows edges from each set corresponding to the 10^th^, 50^th^, and 90^th^ percentiles of *π*_*h*_, and **Fig 3B** (*right*) shows shadows at these same percentiles. Although we observe significant differences in our measure of high spatial frequency content between edges and shadows (**Fig 2C**), we find that when we add this variable to our logistic regression classifier, we do not observe an improvement in discrimination performance. For **oset-1**, a classifier using all three cues can separate occlusions and shadows with an average performance of 66.4% accuracy on the test image sets (non-overlapping with training sets), and 69.7% accuracy on the training sets (**S1 Table**). For **oset-2**, the classifier separates the two categories in the test sets with an average accuracy of 65.9%, with average accuracy of 70.5% on the training sets (**S2 Table**). Similar results were obtained with a second analysis in which both training and test sets were sampled uniformly from the full database, with performance of 68.5% on the test set for **oset-1** and 69.4% on the test set for **oset-2**.

### Image-computable machine learning classifiers

#### Gabor filter bank classifier

Our analysis of image patch statistics demonstrated that simple features like contrast and spatial frequency could separate the two categories well above chance at a resolution of 40x40 pixels. However, this simple set of intuitively defined features does not exhaust all the information potentially available to human or machine observers to separate these two categories. Therefore, to gain additional insight we considered the performance of two biologically plausible image-computable machine learning classifiers.

**Fig 4:**
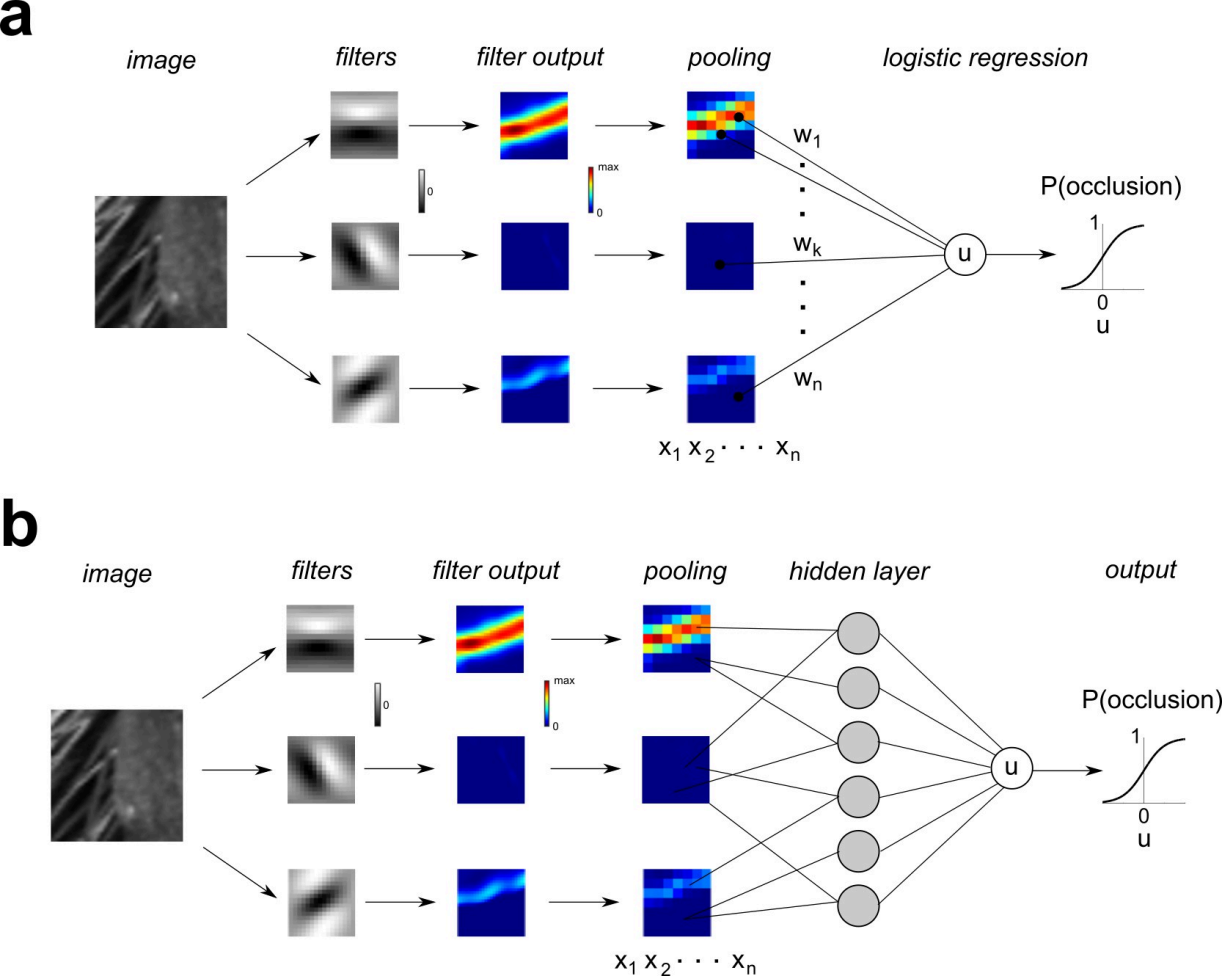
Schematic illustration of Gabor Filter Bank (GFB) and Filter-Rectify-Filter (FRF) classifier models. (**a**) **GFB** model. An image patch is analyzed by a bank of 72 oriented log-Gabor filters covering 3 spatial scales (8, 16, 32 pixels). The rectified filter outputs are downsampled and MAX pooled to obtain a set of regressors *x*_1_,…,*x*_*n*_. Binomial logistic regression is performed to find a set of weights that optimally predict the probability that the patch is an occlusion. (**b**) **FRF** model. This model is identical to the **GFB** model, except the regressors *x*_1_,…,*x*_*n*_ are then passed into a three-layer neural network having *K =* 4, 6, 10 hidden units (*gray circles*) to determine the probability an image patch is an occlusion edge.

The initial representation of local image regions in the visual cortex is computed by the population of V1 simple cells, which are well modeled as a bank of multi-scale linear log-Gabor filters with half-wave rectified outputs. We performed binomial logistic regression on the rectified and down-sampled (MAX pooling) outputs of a bank of physiologically plausible oriented log-Gabor filters [21,22] defined at multiple spatial scales (8x8, 16x16, 32x32), with additional details in **METHODS**. We refer to this model illustrated in **Fig 4A** as the **GFB** (Gabor Filter Bank) classifier, and log-Gabor filters at one spatial scale are illustrated in**. S4 Fig**.

The **GFB** classifier performed significantly better than the classifier defined on contrast statistics (**Fig 2**), and its performance was robust over a wide range of regularization hyper-parameters. Breaking the images into 5 folds of non-overlapping images revealed average test set performance of 78.5% correct for **oset-1** (**S3 Table**), although average test set performance was slightly worse for **oset-2** (70.8% correct, **S4 Table**). Average training set performance was about the same for both sets (**oset-1**: 84.7%, **oset-2**: 82.8%). This poorer generalization ability for **oset-2** may have resulted from the relatively small size of this occlusion set (only 20 images), leading to over-fitting of the training data. When both test sets and training sets were sampled from the entire image database uniformly (with replacement), we found much better model performance on the test sets (**oset-1:** 79.9%, **oset-2:** 80.4%), most likely because they were being trained and tested on similar image patches (**S5 Table**).

**Fig 5:**
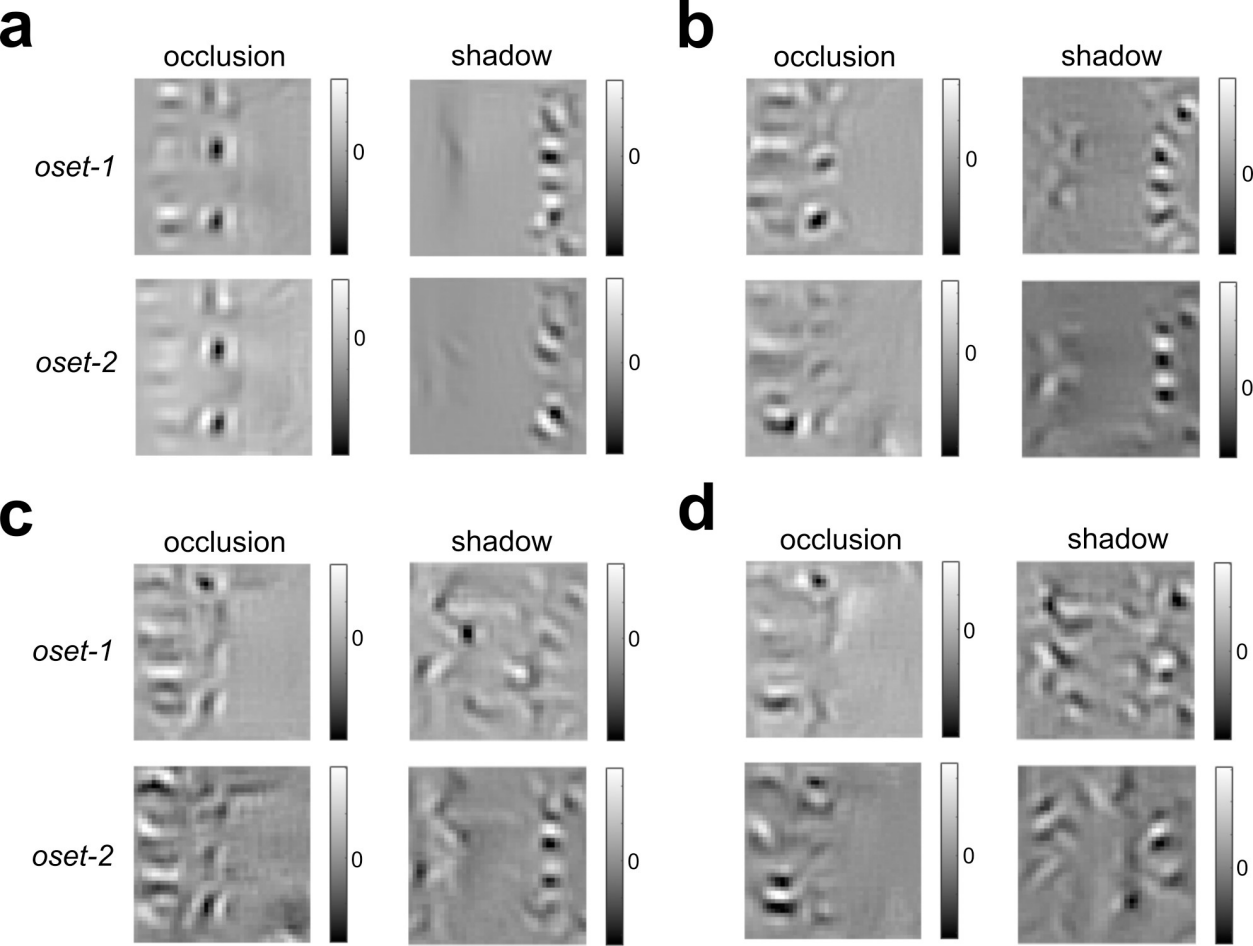
Stimuli that maximize + minimize the occlusion probability predicted by the GFB and FRF models trained to distinguish shadows from occlusions (oset-1, oset-2). (**a**) *Left*: Stimuli obtained via numerical optimization with highest predicted occlusion probability by the **GFB** model. We see consistent results for both training sets, over multiple optimizations. *Right*: Same as left column, but with highest predicted shadow probability by the **GFB** model (equivalently, lowest predicted occlusion probability). (**b**) Same as (a) but for **FRF-4**. (**c**) Same as (a) but for **FRF-6**. (**d**) Same as (a) but for **FRF-10**.

**Fig 6:**
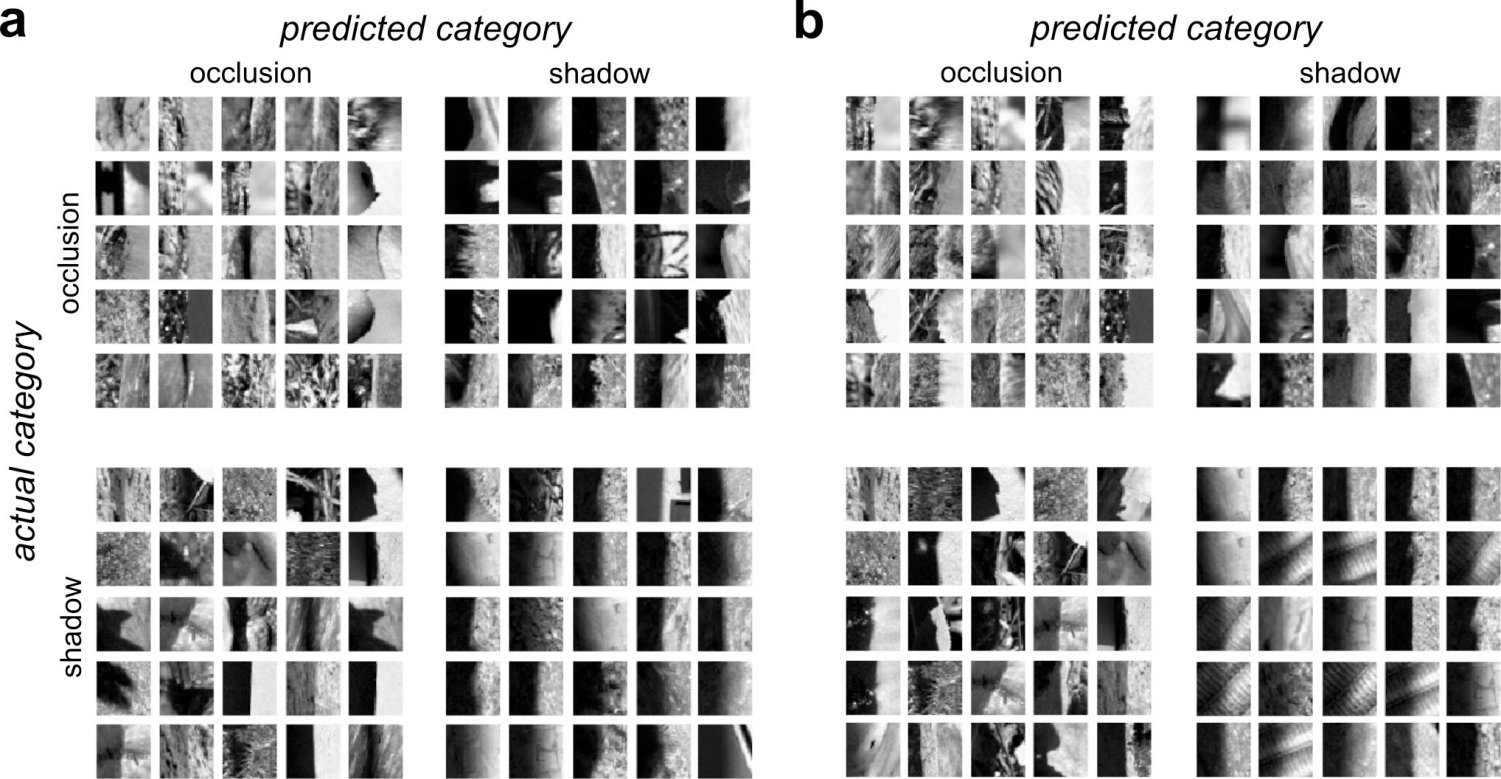
Occlusion and shadow edges with the 25 highest predicted of occlusion and shadow category membership by the GFB and FRF models. Actual category is indicated by the row, predicted category by the column. The upper left and lower right quadrants indicate correct classifications. (**a**) **GFB** model, (**b**) **FRF** model.

To determine the stimulus which would be judged by the **GFB** classifier to be most likely to be classified as an occlusion (or shadow), we numerically found the stimulus to maximize (or minimize) the model output. Unfortunately, neither of these stimuli obtained via optimization visually resembles the actual image patches from either category (**Fig 5A**). However, we do see that the stimulus most likely to be classified as an occlusion has a sharp border, whereas the stimulus most likely to be a shadow is relatively “flat” in the middle. Therefore, we can gain better intuition for what each model is looking for by plotting the edges (of both categories) most likely to be classified as occlusions or shadows, as shown in **Fig 6A**. Each quadrant shows a combination of actual category (vertical dimension) and predicted category (horizontal dimension). We see that the occlusions most likely to be correctly classified as occlusions (*top left*) have low contrast with a sharp boundary between regions and quite often differences in texture on opposite sides of the border. The occlusions most likely to be misclassified as shadows (*top right*) tend to have higher contrast and a somewhat more blurred edge. The shadows misclassified as occlusions (*bottom left*) tend to have lower contrast and/or a sharper edge. And finally, the shadows which are most likely to be correctly classified (*lower right*) have high contrast and blurry edges.

#### Filter-rectify-filter classifier

We next considered the performance of a Filter-Rectify-Filter (**FRF**) style neural network model to classify occlusion and shadow edges (**Fig 4B**). **FRF** models have been long used for modeling texture segmentation [14,16–20,23–25] and are mathematically similar to three-layer connectionist neural networks [26–28]. The first filtering layer of this network was comprised of the fixed, multi-scale Gabor filter bank used in the **GFB** classifier (**Fig 4A**). The rectified, down-sampled outputs of this filter bank were then fed to a population of *K* hidden units (*K* = 4, 6, 10), the rectified outputs of which were linearly combined form a decision variable, which is passed into a sigmoid function to predict the occlusion probability.

When training and test sets were derived from non-overlapping sets of images, adding this hidden layer greatly improved classification performance on training sets, but yielded a more modest improvement for the test sets. For the **FRF** model with 6 hidden units (**FRF-6**), the average performance on training sets approached 90% accuracy (**oset-1**:86.3%, **oset-2**: 89.0%), but the average test set performance (**oset-1**: 80.3%, **oset-2**: 73.8%, **S6** and **S7 Tables**) was only somewhat better than the **GFB** model (**oset-1**: 78.5%, **oset-2**: 70.8%). Similar results were obtained for **FRF-4** (**oset-1** –*train*: 85.2%, *test*: 79.9%; **oset-2** –*train*: 80.5%, *test*: 73.5%; **S8** and **S9 Tables**) and **FRF-10** (**oset-1** –*train*: 87.4%, *test*: 80.5%; **oset-2** –*train*: 91.2%, *test*: 73.6%; **S10** and **S11 Tables**). By contrast, when both the test and training sets were uniformly sampled from all images, for each **FRF** model and each set of stimuli (**oset-1**, **oset-2**), performance on the test sets was commensurate with that on the training sets, approaching 90% (**S5 Table**). This is because in this case the model was being tested on patches which were very similar to those it was trained on.

**Fig 7:**
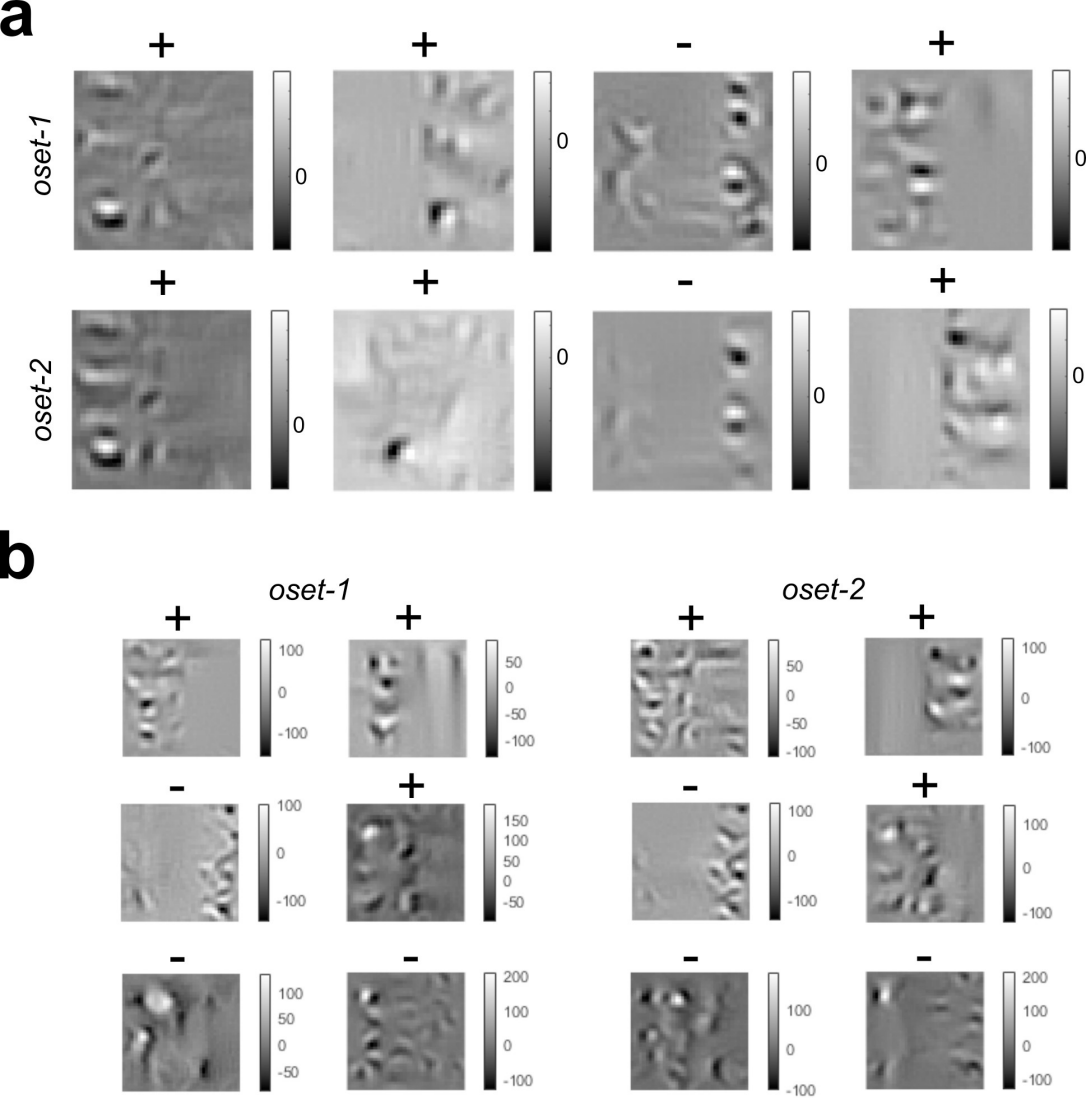
Optimal stimuli for the hidden units in the FRF models with 4 and 6 hidden units trained with both sets of occlusions (oset-1, oset-2). (**a**) **FRF-4** hidden unit receptive fields. Sign of output weight is indicated with a (+) or (-). (**b**) Same as (a) but for **FRF-6**.

As with the **GFB**, numerical maximization (or minimization) of **FRF** model output did not yield stimuli that visually resembled either category (**Fig 5B–5D**). As before, we find the stimulus most likely to be an occlusion has a sharp boundary, whereas a shadow has a gradual boundary. Numerically finding the optimal stimuli for the hidden units in each model revealed that many hidden units seemed to be sensitive to differences in texture on opposite sides of the boundary (**Fig 7**). This is consistent with the idea that texture differences may be important for distinguishing shadows from surface boundaries [20,29].

**Fig 6B** shows the actual and predicted categories of the image patches in **oset-1** the **FRF-6** model predicts to have the highest probability of category membership. We see that as with the **GFB** model, occlusions correctly classified as such (*top left*) tend to have low contrast and sharp borders, and shadows correctly classified as such (*bottom right*) tend to have high contrast and blurred borders. Occlusion patches misclassified as shadows tend to have high contrast and smoother boundaries (*top right*), whereas shadows misclassified as occlusions (*bottom left*) have lower contrast and sharper boundaries.

### Models are sensitive to penumbral blur and texture

#### Tuning for contrast and penumbral blur

**Fig 8:**
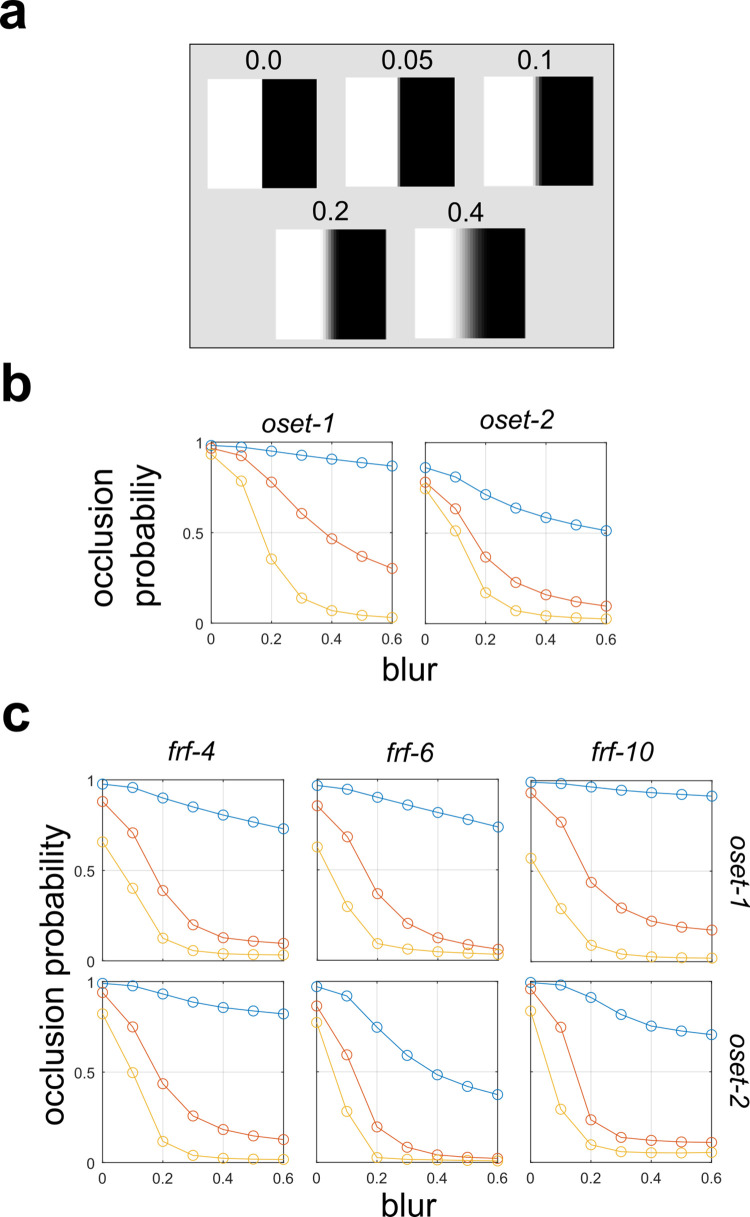
Effects of edge blur on model predictions of edge probability. (**a**) Test image patches with varying levels of blur. (**b**) Gabor Filter Bank (**GFB**) model (see **Fig 4A**) responses to patches with varying levels of blur. Different curves indicate different fixed levels of Michelson contrast (blue = 0.2, orange = 0.4, yellow = 0.6). (**c**) Same as (a) but for Filter-Rectify-Filter (**FRF)** models having varying numbers of hidden units (**Fig 4B**).

In order to understand what image features are being exploited by the **GFB** and **FRF** models to distinguish the image categories, we considered the response of the models to luminance step edges with varying levels of contrast and penumbral blur (**Fig 8A**). **Fig 8B** shows the response of the **GFB** model to step edges with varying degrees of penumbral blur. Different curves denote different levels of Michelson contrast (*c*_*M*_), which is held constant (via numerical image re-scaling) for each stimulus on the curve. We see that increasing the level of penumbral blur decreases the probability that the stimulus is classified as an occlusion edge, or equivalently makes it more likely to be classified as a shadow. Furthermore, we find that as contrast increases, the occlusion probability also decreases, consistent with our analyses shown in **Fig 2**. Similar results were obtained for the **FRF** model, as illustrated in **Fig 8C**. For both models, we see that the sensitivity to both the blur and contrast parameters can be quite dramatic, greatly affecting the edge classification probability.

#### Removal of texture information

**Fig 9:**
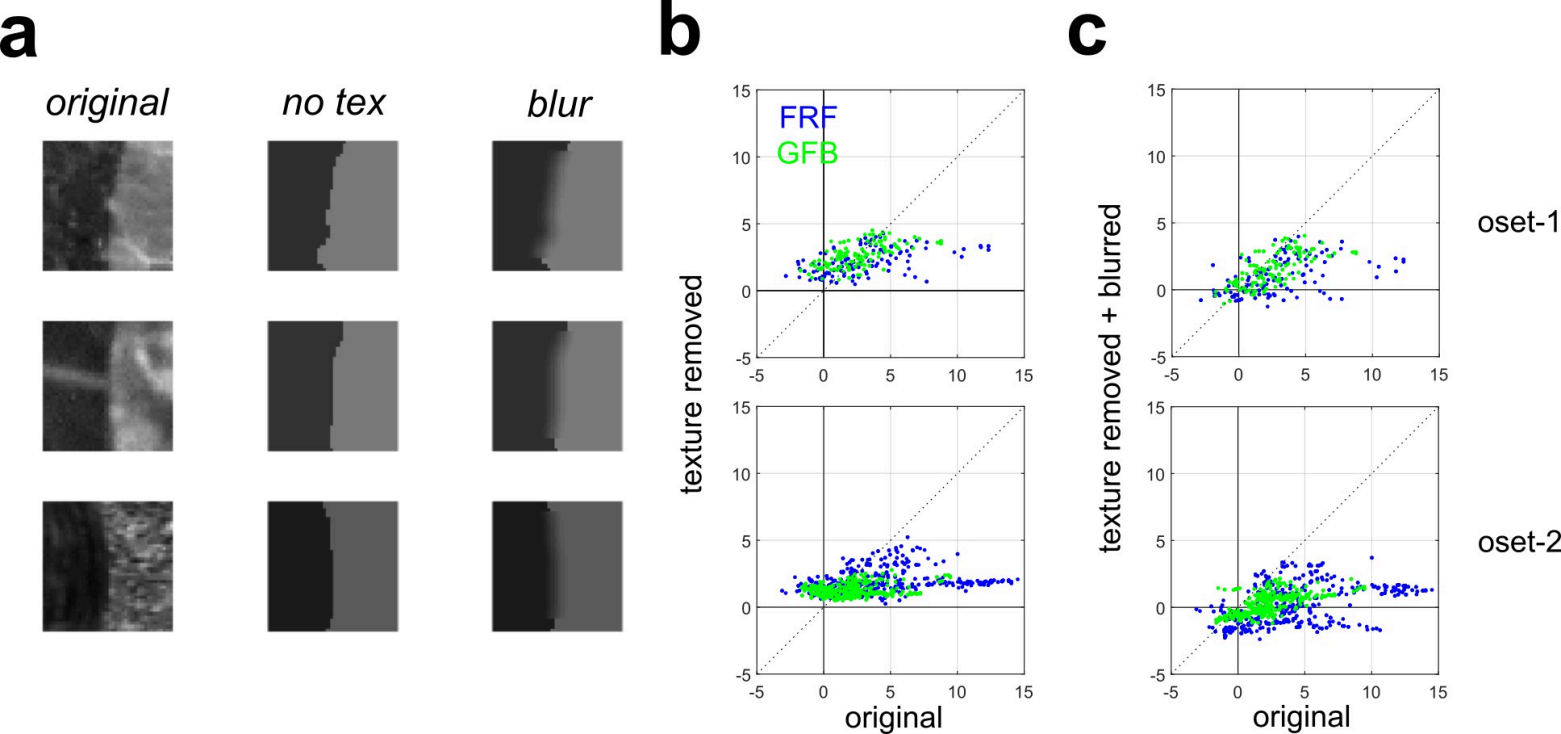
Effects of removing texture information from image patches on responses of the GFB and FRF models. (**a**) *Left*: Original occlusions. *Middle*: Averaging the pixel intensities on each side of the occlusion boundary removes texture information while leaving Michelson contrast unchanged (no tex). *Right*: Blurring the sharp edge created by texture removal (blur). (**b**) Log-odds ratio of being an occlusion for the original images (*horizontal axis*) versus the texture-removed images (*vertical axis*). We see images for which the model predicts a high occlusion probability experience a drastic decrease in occlusion probability when texture is removed. *Top*: **oset-1**, *Bottom*: **oset-2**. (**c**) Same as (b) but for blurred texture-removed patches.

Our analyses of the two models suggests that they may be sensitive to texture differences on opposite sides of the image patch. To test the importance of texture cues more directly, we performed a simple image manipulation procedure to remove texture information from occlusions [3]. Our method simply sets the intensity of each pixel on each side of the boundary equal to the mean value measured from that side. This manipulation leaves the image Michelson contrast (**Eq 3**) unchanged since the mean intensity on each side remains identical. Examples of occlusion patches manipulated in this manner are shown in **Fig 9A** *(middle column*). The output of the **GFB** and **FRF** models is an occlusion probability *p* (with shadow probability being 1−*p*), and can easily be converted to a log-odds ratio (**METHODS**). A positive log-odds ratio indicates that an image patch is more likely to be an occlusion, a negative log-odds ratio indicates more likely a shadow, and zero indicates equal likelihood of each class. **Fig 9B** plots the log-odds ratio for both models (**FRF**: blue, **GFB**: green) for the original occlusion images (*horizontal axis*) and the texture removed images (*vertical axis*) from **oset-1** (*top*) and **oset-2** (*bottom*). Values greater than zero indicate patches that are (correctly) classified as being occlusions, less than zero indicates patches misclassified as shadows. We see patches predicted to have a higher probability of being occlusions experience a much greater reduction in occlusion probability when texture is removed.

For the **FRF** model, statistical testing reveals a significantly positive (Wilcoxon rank-sum test) median value of the difference in log-odds ratios Δ = (original–texture removed) for correctly classified occlusions for both sets (**oset-1**: 0.716, *p* < 0.001; **oset-2**: 2.07, *p* < 0.001). By contrast, for the **GFB** model, for **oset-1** the median value of Δ fails to reach significance (median = 0.165, *p* = 0.138), although it is significant for **oset-2** (median = 1.073, *p* < 0.001). By contrast, for occlusions misclassified as shadows, for the **FRF** model, we find a significantly negative median value of Δ for both sets (**oset-1**: -2.33, *p* < 0.001; **oset-2**: -2.41, *p* < 0.001). For the **GFB** model we also obtain significant negative values (**oset-1**: -2.334, *p* < 0.001; oset-2: -1.861, *p* < 0.001). For each pair of image patches (original and texture-removed), for each model we can compute Δ (Δ_FRF_, Δ_GFB_) and then compute the difference D = Δ_FRF_—Δ_GFB_ to test the hypothesis that removal of texture information has a stronger effect on the responses of the **FRF** model than the **GFB** model (D > 0). In both cases we obtain a median value of D significantly greater than zero (**oset-1:** 0.724, *p* < 0.001; **oset-2**: 0.755, *p* < 0.001), suggesting that the **FRF** model is more sensitive to texture information.

Interestingly, we see in **Fig 9B** that occlusions which were misclassified as shadows (original log-odds < 0) increase their occlusion probability when texture is removed, likely because this operation creates a sharp boundary (**Fig 9A,** *middle*), thereby eliminating the blur which is taken as evidence for a shadow (**Fig 8**). Therefore, as a control, we performed an additional analysis in which after removing the texture we blurred the edge using an 8 x 8 Gaussian filter with a 3-pixel standard deviation (**Fig 9A**, *right*). Blurring was only performed in a central region to prevent edge effects, and the image was re-scaled to have the same Michelson contrast as the other stimuli. Scatterplots analogous to those in **Fig 9B** are shown in **Fig 9C** for these blurred texture-removed image patches. Now we see a general downward shift in the occlusion log-odds predicted by the models, so that some of the blurred texture-removed occlusions are now predicted to be shadows.

For the blurred texture-removed patches, the overall results were similar to the texture-removed patches more generally. For the **FRF** and **GFB** models, we find significantly positive values of Δ for image patches correctly classified as occlusions for both image sets (**FRF**: **oset-1**: median = 1.837, *p* < 0.001, **oset-2**: 3.867, *p* < 0.001; **GFB**: **oset-1**: median = 1.157, *p* < 0.001, **oset-2**: median = 2.03, *p* < 0.001). For incorrectly classified occlusions classified as shadows, while for **oset-1** we observed significantly negative values of Δ (**FRF**: median = -1.002, *p* < 0.001; **GFB**: median = -0.884, *p* < 0.001), this did not obtain for **oset-2** (**FRF**: median = 0.747, *p* = 0.045; **GFB**: median = -0.033, *p* = 0.119). This is to be expected since removing the sharp edge will tend to reduce the occlusion probability. Finally, as before, we find the **FRF** model is more sensitive to these manipulations than the **GFB** model as measured by D = Δ_FRF_—Δ_GFB_ (**oset-1**: median = 0.805, *p* < 0.001, **oset-2**: median = 1.564, *p* < 0.001).

### Psychophysical experiment

#### Individual observer performance

**Fig 10:**
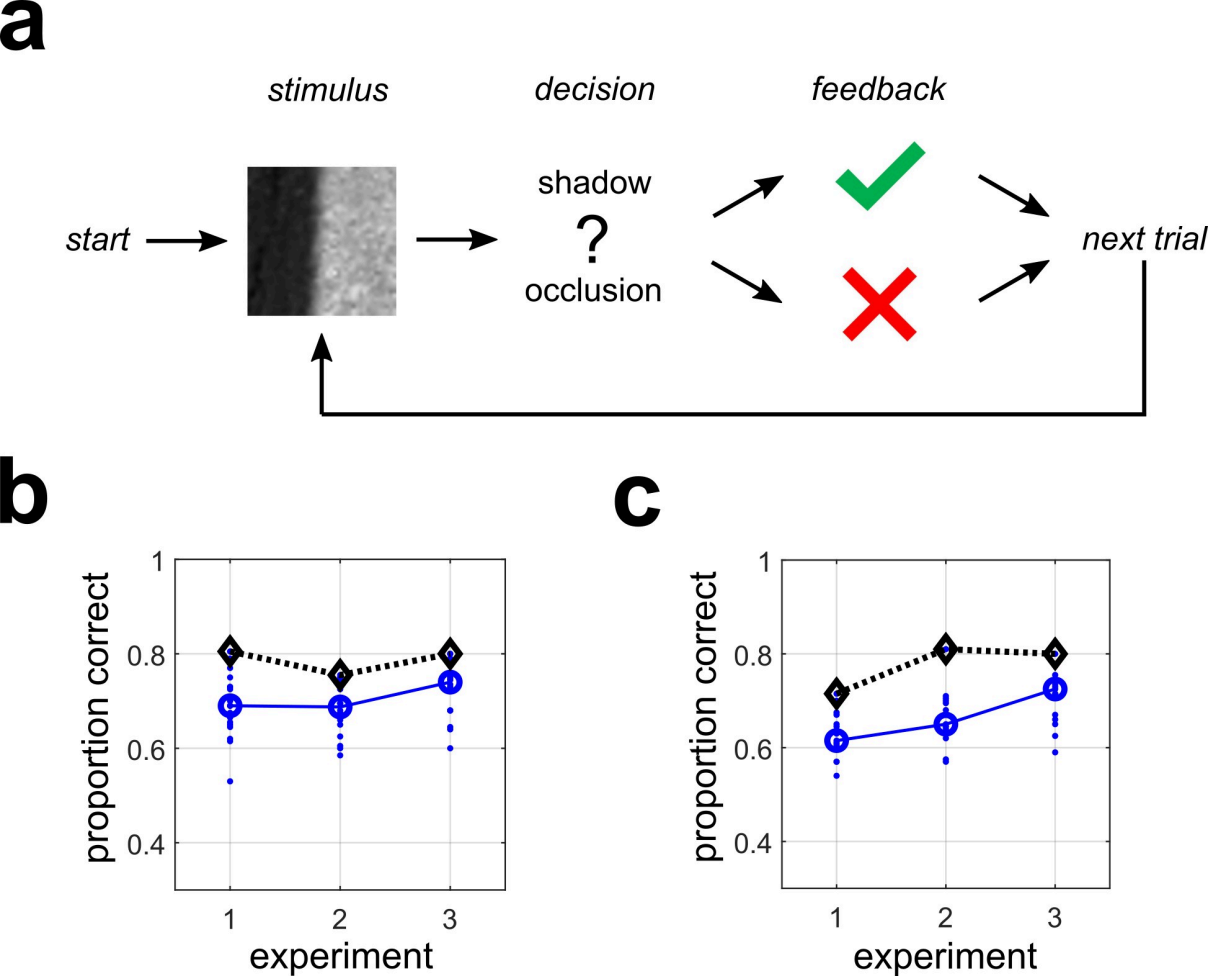
Performance of human observers for each experiment for two different stimulus display methods. (**a**) Schematic outline of a single trial for the psychophysical experiments. For each experiment, human observers classified image patches as either shadows (shown here) or occlusions. (**b**) Performance of all observers (N = 18) on each Qualtrics (**QT)** survey, as measured by proportion correct. Blue dots indicate individual observers. Blue circles indicate the median, and black diamonds indicate maximum performance. (**c**) Same as (b) but for lab (**LB**) experiments (N = 15).

A single-interval forced choice experiment was performed in which human observers classified 40x40 pixel grayscale natural image patches as being shadow or occlusion edges (see **METHODS** for details). This experiment is schematically illustrated in **Fig 10A** and was implemented online using Qualtrics (**QT**) for stimulus presentation and recording responses (**S5 Fig**). The first survey (**QT-1**) was considered a “practice” session, although we found similar results with the “test” surveys (**QT-2**, **QT-3**) and hence included it in our final analysis. All surveys contained 200 stimuli (100 occlusion, 100 shadows) sampled uniformly from the shadow database and **oset-1** (**QT-1**, **QT-2**) or **oset-2** (**QT-3**).

**Fig 10B** shows the performance (proportion correct *p*_*c*_) of all N = 18 observers who completed all three online experiments (with above-chance performance on the final two). We find the median observer (*large blue circles*) had performance of *p*_*c*_ = 0.69 (**QT-1**), *p*_*c*_ = 0.688 (**QT-2**), *p*_*c*_ = 0.740 (**QT-3**), with the best observer attaining performances (*black diamonds*) of *p*_*c*_ = 0.805, *p*_*c*_ = 0.755, *p*_*c*_ = 0.80 respectively. Performance was not significantly correlated across surveys for 2 of 3 possible pair-wise comparisons (**QT 1/2**: *r* = 0.222, *p* = 0.376; **QT 1/3**: *r* = 0.54, *p* = 0.021; **QT 2/3**: *r* = 0.129, *p* = 0.611). Therefore, for many analyses we pooled data across surveys. In these cases, the units of analysis will be referred to as *observer-surveys*.

To validate our online data collection method, we ran a different set of N = 15 participants on these exact same surveys in a standard laboratory setting with calibrated monitors (see **METHODS**). We refer to these surveys as **LB-1**, **LB-2**, **LB-3**. We find similar performance as with the **QT** surveys, with the exception of slightly worse performance on the practice survey (**LB-1**). We find the median observer had performance of *p*_*c*_ = 0.615 (**LB-1**), *p*_*c*_ = 0.65 (**LB-2**), *p*_*c*_ = 0.725 (**LB-3**), with the best observer attaining performances of *p*_*c*_ = 0.715, *p*_*c*_ = 0.81, *p*_*c*_ = 0.80, respectively (**Fig 10C**). Additional analysis of individual observer classification accuracy, broken down by stimulus type, is given in **S1 Text**.

#### Aggregate observer performance across images

In addition to analyzing performance of each individual observer, for each individual image (200 per set, for 3 image sets), we characterized the proportion of observers that classified it as a shadow or an occlusion. Doing this allows us to generate an *aggregate observer* (**AO**) model, which provides a model of average human performance against which the performance of machine classifiers (and other human observers not used to define the model) can be compared. Mathematically, the aggregate observer for K participants making a binary behavioral classification (*b* = 0, 1) of *N* images is given by $A={\left\{\pi_{i}\right\}}_{i=1}^{n}$, where *π*_*i*_ indicates the proportion of observers for which *b*_*i*_ = 1. The **AO** model provides a statistical model of average human performance on the task. One can generate simulated datasets stochastically using the **AO** by generating response *b*_*i*_ = 1 to stimulus *i* with probability *π*_*i*_, and response *b*_*i*_ = 0 with probability 1−*π*_*i*_. Likewise, one can generate a stimulated dataset deterministically from the **AO** model by setting *b*_*i*_ = 1 if *π*_*i*_≥0.5, and *b*_*i*_ = 0 otherwise.

**Fig 11:**
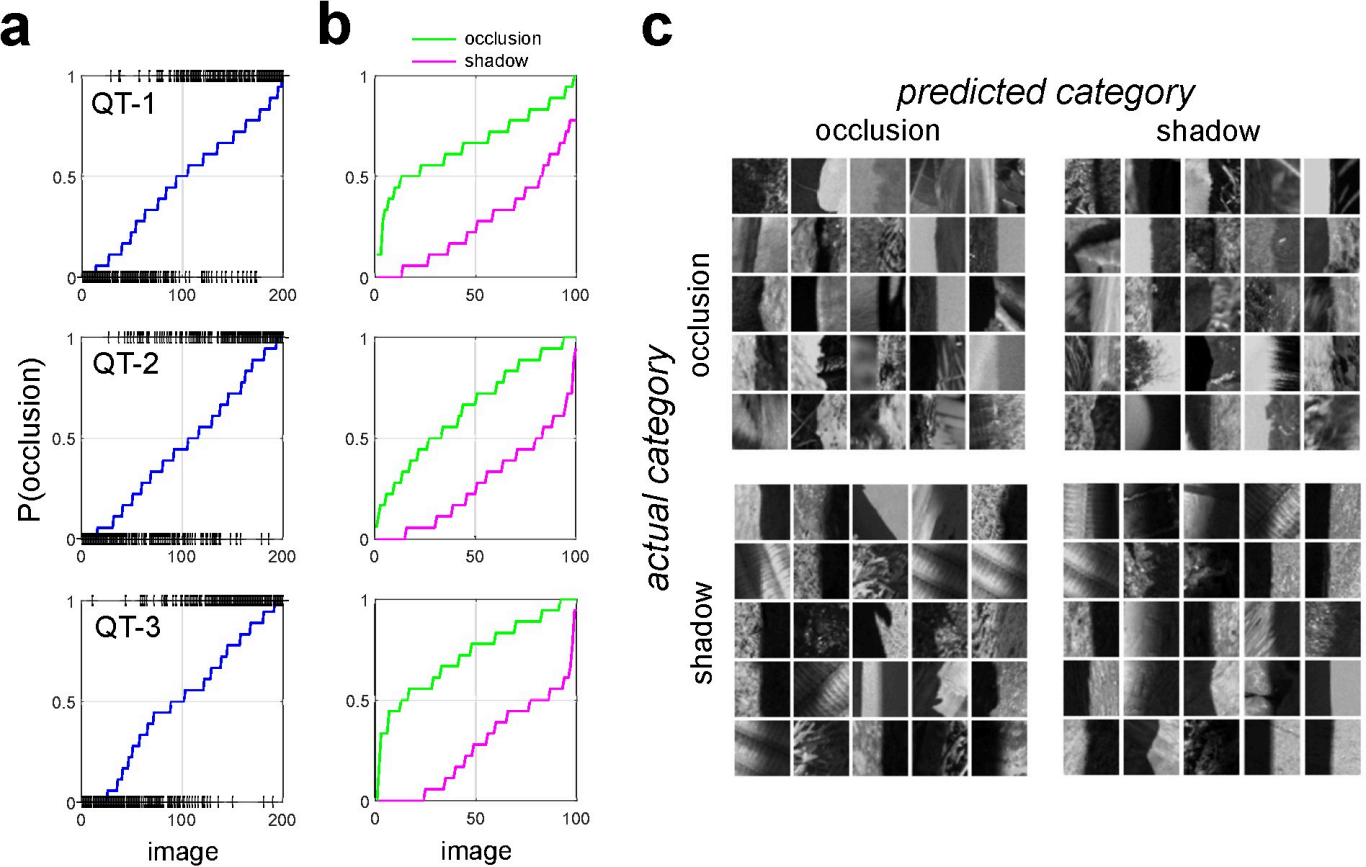
Human performance on individual images. (**a**) Predicted probability of being an occlusion edge by the **AO** (*blue curve*). Image patches are sorted by their probability. Actual category is indicated by a (+). (**b**) Occlusion probability predicted by the **AO** for each occlusion edge (*green curve*) and each shadow edge (*magenta curve*), sorted by occlusion probability. (**c**) Patches of each actual category which are most likely classified by human observers as the predicted category for **QT-2**.

**Fig 11A** shows for the **QT** surveys the probability that each stimulus is classified as an occlusion by the **AO** (blue curves), with actual stimulus categories indicated by a black (+) symbol. We see that the overwhelming majority of the stimuli with low occlusion probability are shadows, and most stimuli with high occlusion probability are occlusions, with little bias in either direction. **Fig 11B** breaks down these plots by the actual category of the image. Analogous plots for the **LB** surveys are shown in **S6 Fig**. **Table 1** indicates the confusion matrices for each **QT** survey obtained from the **AO** using a deterministic rounding rule that sets *b*_*i*_ = 1 if *π*_*i*_≥0.5, and *b*_*i*_ = 0 otherwise. Table 2 indicates these same confusion matrices for the **AO** derived from the **LB** surveys.

**Table 1:** AO confusion matrix for all QT experiments.

	QT-1		QT-2		QT-3	
	predicted		predicted		predicted	
**actual**	*occlusion*	*shadow*	*occlusion*	*shadow*	*occlusion*	*shadow*
*occlusion*	88	12	74	26	88	12
*shadow*	19	81	21	79	24	76

**Table 2:** AO confusion matrix for all LB experiments.

	LB-1		LB-2		LB-3	
	predicted		predicted		predicted	
**actual**	*occlusion*	*shadow*	*occlusion*	*shadow*	*occlusion*	*shadow*
*occlusion*	69	31	73	27	83	17
*shadow*	27	73	22	78	19	81

For each experiment, we tested whether the proportions of errors were biased in one or another direction, with the null hypothesis being that the two proportions were equal. A binomial proportion test revealed no significant difference for **QT-1** (*p* = 0.281, N = 31), **QT-2** (*p* = 0.559, N = 47), or **QT-3** (*p* = 0.066, N = 36). Similarly, we observed no difference for **LB-1** (*p* = 0.694, N = 58), **LB-2** (*p* = 0.566, N = 49), or **LB-3** (*p* = 0.867, N = 36). Therefore, the **AO** is just as likely to misclassify occlusions as shadows as it is to make the opposite error.

### Comparison with machine classifiers

For each image, the aggregate observer model (**AO**) specifies a probability that the image is an occlusion, much in the same manner as the machine classifiers. In order to see if images which were more likely to be categorized as occlusions by the **AO** were also more likely to be categorized as such by various machine observers, we developed a simple analysis procedure, which we now describe. Briefly, each of these machine classifiers outputs the probability *p*_*i*_ of image *i* being an occlusion. As detailed in **METHODS**, this can be converted to a log-odds ratio, which unlike raw probabilities, (**S7 Fig**) enjoys an approximately normal distribution, with a log-odds of zero corresponding to equal likelihood of each category.

**Fig 12:**
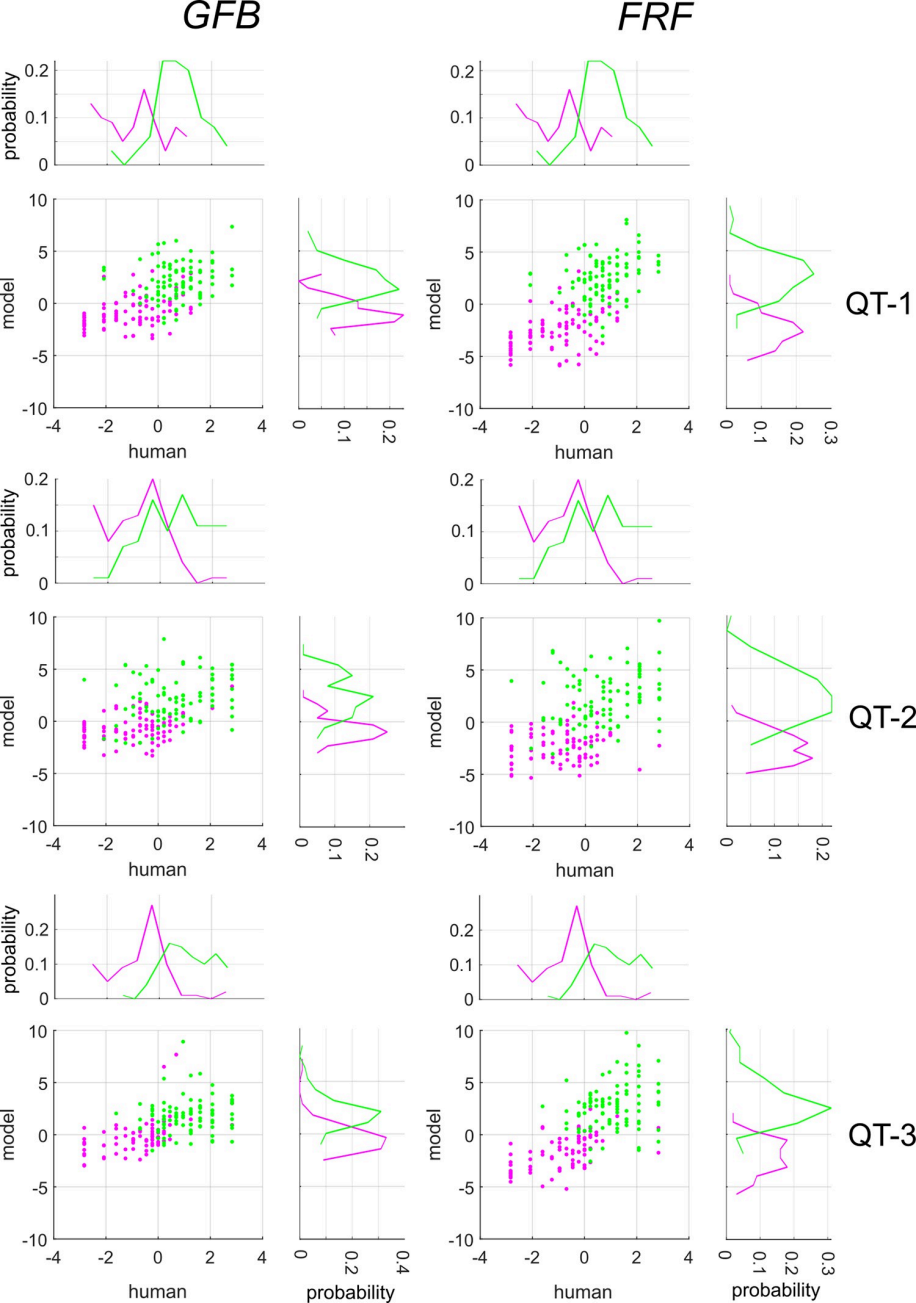
Log-odds ratios of human observers (AO, horizontal axes) and GFB, FRF models (vertical axes) for each image for all three surveys. Magenta symbols indicate image patches that are shadows, green symbols indicate occlusions. On the margins of each scatterplot we show the distributions of the log-odds ratios for each image category.

By correlating the log-odds ratios over the set of images, we can determine the extent to which various machine classifiers exhibit agreement with human observers. **Fig 12** shows scatter-plots of the log odds ratios obtained from human and machine observers applied to all images in each survey (**QT-1**, **QT-2**, **QT-3**) for both the **GFB** (*left column*) and **FRF-10** (*right column*) model. We observe for both models strong, positive correlations between the log-odds ratios obtained from the **AO** and the models, as summarized in Table 3. We made use of Spearman rank-order correlation *ρ* instead of Pearson’s *r* because some images were classified by all observers as shadows or occlusions, so that *p*_*i*_ = 0 or 1, making *σ*^−1^(*p*_*i*_) is undefined.

**Table 3:** Spearman correlation between the log-odds ratio obtained from the aggregate observer (AO) and the machine classifiers over the N = 200 images in each survey. All values are significant with *p* < 0.001.

survey	GFB	FRF
QT-1	0.604	0.696
LB-1	0.475	0.545
QT-2	0.568	0.587
LB-2	0.610	0.627
QT-3	0.697	0.775
LB-3	0.743	0.767

We found that for all surveys a larger Spearman correlation between the **FRF** and the **AO** (Table 3). To test whether there was a stronger correlation between the **GFB** and **FRF** model and human behavior, we computed the Spearman correlation between the log-odds ratios from the **FRF** and **GFB** model and the **AO** for N = 1000 bootstrapped samples. Pooling all three **QT** surveys revealed a significantly larger correlation between the **FRF** and **AO** (*ρ* = 0.692) than the **GFB** and **AO** (*ρ* = 0.624), with a median difference Δ*ρ* = 0.068 and the 95% CI of the difference [0.022, 0.118] not containing zero. Similar results were obtained with **FRF-6**, with median difference (pooling across surveys) between the correlations of Δ*ρ* = 0.073 and 95% CI of the difference [0.028, 0.123] not containing zero.

The results obtained from the **LB** surveys (**S8 Fig**) exhibited effects in the same direction as the **QT** surveys (median Δ*ρ* = 0.039). Pooling all **LB** surveys yields a larger correlation between **AO** and **FRF** (*ρ* = 0.652) than **GFB** (*ρ* = 0.614), although the 95% CI of the difference [−0.011, 0.092] contains zero, and therefore this difference narrowly misses statistical significance. However, taken as a whole, this analysis suggests that the **FRF** model is at least as good, and possibly somewhat better, than the **GFB** model at capturing whatever aspects of the image humans are utilizing. Additional comparisons of model and human performance were performed by comparing patterns of misclassification between the **AO** and machine classifiers, and computing decision-variable correlations [30] between individual observers and machine classifiers. On the whole, these analyses also demonstrate a better agreement between the **FRF** model and human performance. Details may be found in **S1 Text**.

## Discussion

### Summary of contributions

Here we analyzed the statistical properties of two important classes of luminance edges: (1) occlusion edges, and (2) shadow edges. Distinguishing these two possible edge causes has important implications for understanding perceptual organization, as one kind of edge indicates a surface boundary, whereas the other indicates a change in illumination. Following previous work [3,7], we measured basic contrast statistics from each category, demonstrating that occlusions exhibit both the highest observed contrasts, as well as the lowest contrasts. We also measured spatial frequency properties of each image category, demonstrating more high spatial frequency content in occlusion edges than shadows. V1 neurons are known to be sensitive to both spatial frequency and contrast [31,32], so relevant information for discriminating these edge categories is potentially extracted at the earliest level of cortical processing.

Implementing a simple Gabor Filter Bank (**GFB**) model inspired by V1 receptive field properties, we demonstrated that at a resolution of only 40x40 pixels these two edge categories can be distinguished at nearly 80% accuracy. Furthermore, we demonstrated that this model exhibits strong tuning to penumbral blur, an important shadow cue [6,8,33–35]. To investigate the performance of models which can also take texture into account, we demonstrate that a Filter-Rectify-Filter (**FRF**) model, commonly used to model second-order vision [14,16–18,25], can perform at greater than 80% accuracy.

In order to determine to what extent these models are predictive of human performance when applied to the same stimuli, we ran a simple psychophysical experiment (using both online and laboratory methods) on a large number of observers. We found that although each model (**GFB**, **FRF**) exhibited performance commensurate with the best observers, when applied to the same stimuli there was a better agreement on an image-by-image basis between the human observers and the **FRF** model. Since the **FRF** model exhibits greater sensitivity to texture differences, this suggest that human observers may be making use of this cue in their classifications. This would certainly be consistent with the findings of [12], who observed an improvement in human performance in a shadow/material classification task with increasing aperture size, which increases the availability of texture cues.

### Relationship to other work

Most psychophysical studies concerning shadows have investigated the essential role that shadows play in three-dimensional scene interpretation [5,6,35–37]. By contrast, to our knowledge, only two previous psychophysical studies directly speak to the issue of what locally available information can be used to distinguish shadows from other edge causes like occlusions [7,12].

A previous investigation [7] characterized the statistical properties of achromatic occlusion edges and non-occlusion edges, a “catch-all” category including shadows, material changes, and surface orientation changes. Although they used local analysis windows similar in size to our own (they used rectangular windows of 81x40 whereas we used square 40x40 windows), the images which they analyzed were at high resolution (1920x2560) as opposed to the lower resolution (576x786) images we analyzed. The consequence of this is that their analysis was more “local” than our own, with their smaller dimension (40) comprising only 1.56% of the larger dimension (2560) of the image patch, whereas our 40x40 patches subtended 5.08% of the larger dimension (786). Finally, their edges were initially identified using a Canny edge detector algorithm before being classified as occlusions or non-occlusions by human observers. As they note, this process biases their samples towards edges containing stronger luminance differences, although they also analyzed a smaller set of hand-labeled edges and obtained similar results.

In their paper, they demonstrated that occlusions had higher contrast, on average, than non-occlusions. We found that when the comparison was restricted to occlusions and shadows, both the highest contrast patches as well as the lowest-contrast patches were occlusions (**Fig 2**). Furthermore, when we analyzed occlusions from the subset of 19 images in **oset-1** that were also used in their study, the results were consistent with our general findings for both **oset-1** and **oset-2** (**S2 Fig**). Therefore, it is unlikely that this discrepancy with our results arises from their use of a more limited occlusion image set (N = 38 images versus N = 100). It is important to note that there are two additional key differences which make direct comparison difficult: (1) We did not consider any non-occlusions apart from shadows in our analysis, and (2) They did not break down their non-occlusion stimuli into different categories in their analysis. Thus, it is possible that their finding that non-occlusions had lower contrast than occlusions would not have held if they had restricted themselves to only consider shadows.

Another interesting point of contact with [7] is that in their study they plotted the luminance profiles of different categories of edges, including occlusions, non-occlusions, and shadows. They note a more gradual change in luminance for non-occlusions, including shadows, than for occlusions. All the image-computable models trained to distinguish shadows from occlusions exhibited tuning to penumbral blur, with increased blur leading to higher probability of an edge being classified as shadow (**Fig 8**). Previous experiments suggest that humans are adept at distinguishing sharp from blurred edges [13,38], and our analysis suggests this ability may help solve an important problem in natural scene analysis.

Another closely related study which served as a strong motivation for the present work is a recent study [12] demonstrating the importance of chromatic cues for accurate edge classification as luminance or material. One interesting finding to arise from their study is that performance in the task for human subjects increased with edge size, whereas a similar improvement in performance was not observed with machine classifiers defined using luminance and chromatic cues. A reasonable interpretation of this result is that human observers were able to exploit textural cues which were not incorporated into the machine classifiers. Consistent with this interpretation, the present work also demonstrates that texture cues can be potentially exploited for distinguishing shadows from occlusions. Training “Filter-Rectify-Filter” (**FRF**) style classifiers revealed hidden units exhibiting sensitivity to differences in texture (**Fig 7**), and removing texture from the image patches lead to a drastic reduction in occlusion probability for those images most likely to be occlusions (**Fig 9**). The utility of texture for distinguishing changes in illumination from changes in material is relatively unsurprising, as different surfaces usually have different textures. However, what is perhaps quite surprising is our finding of just how much information highly localized texture differences (40x40 pixels) can contribute.

### Limitations and future directions

The present study has several limitations, each of which provides a fruitful avenue for future work. Perhaps the most serious limitation is that we only consider the discrimination between shadows and surface boundaries, the vast majority of which are depth occlusions. However, as noted by others [7], there are a vast number of non-shadow edge causes in natural images, including changes in reflectance (for instance, paint color), changes in texture, depth occlusions, juxtapositions of different surfaces, changes of surface orientation in 3-D with respect to an illuminant, and specular reflections. Previous research which motivated the present study [12] tested the ability of observers to distinguish between “changes in material” and “changes in illumination” (i.e., shadows). Their “changes in material” category certainly encompasses our surface boundaries, but also contains several edge categories which were not represented in the present study, for instance changes in reflectance on a surface (paint color) or changes in texture or patterning.

Our focus on surface boundaries was partly practical, as we have access to a large database of surface boundaries (mostly depth occlusions) taken from the same calibrated image database as our shadows [9]. It is of great interest for future work to develop hand-labeled databases of non-occlusion material changes to investigate edge classification more broadly. This could be done by expanding the current databases, which are freely available. However, there is a very strong principled reason to focus on surface boundaries as well. Assuming that the goal of mid-level vision is to parse the image into regions representing distinct surfaces [1], then misclassifying a shadow as a surface boundary is potentially much more consequential an error than misclassifying a shadow as a reflectance change on a surface. Therefore, we feel that apart from the issue of practicality, there is a very good justification for the focus on distinguishing cast shadows from occlusions and surface boundaries more broadly.

This concern notwithstanding, there are several minor limitations which merit further mention. Firstly, as in many other studies of natural perception and computer vision [2–4,39–41], we rely on image features annotated by human observers in a relatively small number of visual scenes. Therefore, the validity of such analyses relies on the assumption that the image sets chosen, and the region within these images which are annotated by human observers, are indeed representative of the natural visual environment.

Secondly, we focus here only on achromatic cues for shadow/occlusion classification, as previous work has rigorously investigated color and its importance for shadow edge/material edge classification [12]. It would be of great interest for future work to incorporate color into our neural network analyses. Given the potential utility of chromatic information for distinguishing illumination from material [9,11], we might expect to find hidden units which learn to detect different colors on opposite sides of the boundary. Indeed, studies of deep neural networks trained on RGB images for classification tasks have been shown to learn filters specialized for detecting color edges while being insensitive to luminance [42,43]. Despite the obvious interest of including chromatic cues, our work clearly demonstrates that they are by no means necessary for accurate shadow edge classification from local cues. A simple perusal of the grayscale images in **Fig 1A** serves as proof in fact that while most certainly useful, chromaticity is by no means necessary for edge classification, and perceptual organization more broadly. It remains for future work to determine the extent to which human observers performing the same task as our machine classifiers can exploit these various achromatic cues, and to what extent their performance on an image-by-image basis is consistent with that of the machine classifiers.

Our finding that local cues can be fruitfully exploited to assist global scene interpretation is consistent with many previous studies on natural edge detection [3,39,41,44], classification [7,12,45], and figure-ground assignment [46,47]. However, one major limitation of this kind of psychophysical study is that natural images cannot be easily manipulated in order to demonstrate which exact features are being used by human observers to solve the natural vision task under consideration. Nevertheless, it is certainly not impossible to modify natural stimuli or to create synthetic versions of naturalistic stimuli which permit factorial manipulation of individual image parameters.

In the current work, we demonstrate positive correlations on an image-by-image basis between two models which exhibit sensitivity to penumbral blur and texture cues, suggesting these cues may be important for human perception. However, unless we are able to manipulate these cues independently, we cannot understand how they combine. Therefore, it is of great interest for future work to create synthetic stimuli in which two textures whose degree of similarity can be systematically modified are quilted together to form a boundary [16,18]. To this texture boundary one can add a luminance step whose contrast and sharpness (blur) can also be varied parametrically. One might expect that stimuli with large texture differences and a sharp luminance step would be more likely to be classified as occlusions, whereas stimuli with small texture differences and a blurry luminance step will be classified as shadows. By independently varying these two parameters, one can in principle understand the summation rules by which these cues combine, as well as their relative weighting [48–50]. Conducting these experiments is of great interest for future psychophysical studies, and we hope that the present paper serves as inspiration.

## Methods

### Ethics statement

For all human subject experiments, we obtained prior approval from the Institutional Review Board (IRB) of Florida Gulf Coast University (IRB Protocols 2014–01, 2021–78). Before each experiment, participants were required to review and accept an informed consent form, in accordance with the Declaration of Helsinki.

### Shadow and occlusion databases

#### Image sets and hand-labeling procedures

We obtained a set of 47 shadow images (786x576 resolution) taken from the McGill calibrated color image database [9]. The set of 47 images contained both natural and manmade objects. All selected images contained clear figure-ground organization and detectable shadow boundaries. Authors CD and BG with two other undergraduate researchers (LA, MD) used the image manipulation program GIMP (https://www.gimp.org/) to label the most noticeable shadows within the set of images. Since observers were not explicitly instructed to be exhaustive in their labeling, it is possible that some perfectly valid shadows may have been missed (**Fig 1A**, left image). Each student was instructed to label the shadows individually, so their labels reflected their independent judgment. Examples of images from our shadow database (**shad**) are shown with the labeling of the shadow contours in each image by one observer (MD) shown in **Fig 1A** (*top*). A binary union of the labels from all 4 observers is shown in **Fig 1A** (*bottom*).

Image patches containing surface boundaries were obtained from two sets of images. One was a previously developed image database [3], also derived from a subset of 100 images (786 x 576) from the calibrated McGill image set. We refer to as this set of images as occlusion set-1 (**oset-1**). However, despite its name, it is important to note that not all the labeled surface boundaries are true depth occlusions, with several labeled edges being better described as the juxtaposition of two different surfaces in the same depth plane, for instance the border between a sidewalk and grass (**S1 Fig**). Therefore, although this database exhibits a strong bias towards depth occlusions, several the labeled surface boundaries could also be more accurately labeled as “material changes” [12]. As with the shadow database, since observers were not explicitly told to be exhaustive in their labeling, many perfectly valid surface boundaries may not have been labeled.

One potential limitation of **oset-1** is that the images in this set have almost no overlap with our set of 47 shadow images. Therefore, it at least possible that any statistical differences observed between shadow edges and occlusion edges could be a function of there being different surfaces present in the two image sets. Therefore, we developed a second occlusion database by having three of the four observers who labeled the shadow contours also label the occlusions in a subset of 20 images taken from our larger database of 47 shadow-labeled images. We refer to this second database of occlusions taken from a subset of our 47 shadow images as occlusion set-2 (**oset-2**), and the same caveats outlined above for **oset-1** apply to **oset-2**. Two example images from this set (**oset-2**) with labeled occlusions are shown in **Fig 1B** (*top*), together with a binary label of the occlusions from three observers (**Fig 1B**, *bottom*). Our hand-labeled shadow database and both occlusion databases are freely available at: https://www.fgcu.edu/faculty/cdimattina/.

#### Image patch extraction methods

Both occlusion edges and shadow edges were extracted from the image as described in [3]. Briefly, random pixels labeled as edges by a single observer were chosen, cycling through the set of observers who labeled each image. To ensure patches where the boundary separates two regions of approximately equal size, with no extraneous edges, a patch centered on an edge was only accepted for inclusion if the composite edge map (pooled over all observers) for the candidate patch comprised a single, connected dividing the patch into two regions, the smaller of which comprised at least 35% of the pixels. This procedure excludes T-junctions or highly convex edges, yielding mostly smooth edges as is evident from the image patches of both categories (occlusions, shadow) shown in this paper. We focused our analyses on 40x40 image patches, which in a 786x576 image constituted roughly 5% of the larger dimension, ensuring our analysis focused on relatively local cues. Representative patches from each set are shown in **Fig 1C**.

### Analysis of image patch statistics

#### Contrast statistics

Previous investigations have demonstrated interesting differences in the statistical properties of occlusion patches and non-occlusions (including shadows) and shown that these statistics can be utilized to reliably discriminate these two categories [7,12]. Therefore, we measured some of these same quantities from our image patches at 40x40 resolution to provide basic descriptive statistics for the shadow and occlusion edges in our databases, and to determine the extent to which such simple statistics could be reliably exploited for image classification purposes.

From both the shadow and occlusion edges, we measured two quantities from linearized RGB versions of the images that were first converted to grayscale: (1) RMS contrast, and (2) Michelson contrast. Conversion to grayscale was accomplished using the standard formula

I=0.2989*R+0.5870*G+0.1140*B. (1)

The RMS contrast of the grayscale image *I* is defined as

$$c_{RMS}=\frac{\sigma }{\mu },$$ (2)

where *σ* is the standard deviation of the pixel intensities, and *μ* is the mean pixel intensity. Since our selection method only chose image patches which could be cleanly divided into two regions of approximately equal size (see [3] for details), we were also able to measure the Michelson contrast, which is defined as

$$c_{M}=|\frac{\mu_{2}-\mu_{1}}{\mu_{2}+\mu_{1}}|,$$ (3)

where *μ*_1_, *μ*_2_ denote the mean pixel intensity in each of the two regions.

Making use of the MATLAB machine learning toolbox function fitclinear.m, we trained a logistic regression classifier [27] on these two variables to see to what extent they could distinguish the two image categories. This was done in two different ways. In the first analysis, we broke each image set (shadows, occlusions) into 5 blocks of non-overlapping images. The images in **oset-1**, **oset-2** and **shad** comprising each block are listed in **S1** and **S2 Tables**. We then trained the classifier on 4 of the blocks (*training sets*, 50,944 images) while testing on the 5^th^ (*test set*, 12,736 images). We obtained an estimate of the classifier ability to generalize to novel images by averaging test set performance over all 5 choices of test set. In a second analysis, we trained on 16,000 images sampled uniformly (with replacement) from all the images. We then tested classifier performance on a test set comprised of 4000 images also sampled uniformly with replacement from all the images.

#### Spatial frequency analysis

In addition to contrast statistics, another potential cue for distinguishing edges caused by shadows from those caused by occlusion boundaries is spatial frequency content. Due to penumbral blur, many (but not all) shadows exhibit a gradual luminance change at their boundary [8], whereas occlusions typically give rise to a relatively sharp luminance transition. Furthermore, psychophysical experiments using classification image techniques have demonstrated that human observers can reliably discriminate sharp luminance edges from blurry ones [38], suggesting penumbral blur can be potentially employed for shadow identification. We applied the standard FFT to the 40x40 image patches from both categories, which were first pre-processed to have zero mean and identical energy. We then quantified the proportion of stimulus energy in various high spatial-frequency regions of the spectrum. This was accomplished by taking the rotational sum of the power spectrum and then taking the ratio *π*_*h*_ of power above 10 cycles/image to total stimulus power (Nyquist frequency for 40 x 40 images is 20 cycles/image). This parameter *π*_*h*_ was combined with the two contrast parameters *c*_*RMS*_, *c*_*M*_ to reimplement the logistic regression classifier analysis described in the previous section with a larger feature set.

#### Image-computable classifier analyses

Three categories of image-computable machine classifiers were implemented in Python using Keras and Tensorflow [51]. To align the images to improve classification accuracy, image patches were rotated into a vertical orientation, and reflected into sine phase (if necessary) so that the left side was always darker than the right side.

As a robust test of model generalization ability, image-computable models were trained on 4 blocks of images (training sets, 50,944 images) and tested on a 5^th^ block (test sets, 12,736 images) having no overlap with the training sets. In this analysis, the same number of images were used for training and testing both the **FRF** and **GFB** models. This was repeated for a wide range of regularization hyper-parameter values (log_10_ *λ* = −3,−2,−1,0,1,2,3), and we report the average performance over 5 test sets for the best hyper-parameter. In our analyses, we found that for all models the performance was generally quite robust over a large range of hyper-parameter values.

For exploring model sensitivity to blur and texture, and comparing model performance with human performance, we trained the models using our full set of images. For this analysis we trained models on sets of 16,000 (**GFB** model: 8.88 samples/parameter) or 56,000 (**FRF** models: 3.11–7.77 samples/parameter) images sampled uniformly with replacement from the entire dataset, and tested the models on much smaller sets of 4000 images, also sampled uniformly from the entire dataset.

#### Gabor filter bank classifier

The Gabor Filter Bank (**GFB**) model is illustrated schematically in **Fig 4A**. In this model, a fixed set of multiscale log-Gabor filters (8, 16, 32 pixels) [21,22] was convolved with an input image (40x40 pixels). Filter parameters were set to yield a spatial frequency bandwidth of roughly 1.5 octaves, typical of V1 cortical neurons [52]. Links to MATLAB code may be found in [21]. At each scale, the set of oriented filters includes 6 orientations, 2 phases (even/odd), and 2 contrast polarities (+/-), for a total of 24 oriented filters (**S4 Fig)**. The outputs of these filters were half-wave linear rectified (*relu*) and then down-sampled from 40x40 to 5x5 using MAX pooling [28]. As larger filter sizes (16, 32) analyze over-lapping regions of the 40x40 image, the responses within each 5x5 MAX pooling array at these scales will generally exhibit correlations. Binomial logistic regression [27] was performed on these 1800 pooled filter responses as predictor variables with stimulus category (0 = shadow, 1 = occlusion) as the response variable.

#### Filter-rectify-filter classifier

Finally, we implemented the Filter-Rectify-Filter (**FRF**)-style model often used to account for texture segmentation [14,15]. This model is illustrated in **Fig 4B**. As with the **GFB** model, the image was initially convolved with a set of fixed Gabor filters, and the linearly rectified (*relu*) outputs downsampled using MAX pooling. However, these down-sampled outputs were fed into a three-layer neural network classifier having N = 4, 6, or 10 hidden units with *relu* gain. For analyzing the effects of texture removal on the **FRF** model (**Fig 9**), occlusions having vertical or near-vertical orientation were chosen from each image database from a set of images not used for model training. Texture information was removed from these patches using the same method as in our previous work [3], by simply setting the intensity to each pixel in a region equal to the mean intensity over the region. Image patches manipulated in this manner are shown in **Fig 9A**. This manipulation removes all texture cues while leaving Michelson contrast (**Eq 2**) unchanged.

#### Conversion from probabilities to log-odds

Each of these machine classifiers at its final stage computes an intermediate variable $u_{i}=\ln\frac{p_{i}}{1-p_{i}}$ (the log-odds ratio) which is them passed through a monotonic sigmoidal non-linearity to compute a probability *p*_*i*_ of image *i* being an occlusion. By applying the inverse sigmoid function *u*_*i*_ = *σ*^−1^(*p*_*i*_) to the probability computed by the model, we recover the value of the log odds ratio, which unlike the occlusion probabilities enjoys an approximately normal distribution. Note that *σ*^−1^(0) and *σ*^−1^(1) are not well defined (being −∞ and +∞, respectively).

### Psychophysical experiment: Qualtrics

#### Participants

Participants consisted of undergraduate students attending Florida Gulf Coast University (FGCU) enrolled in Psychology courses PSB-4002 (“Brain and Behavior”) and EXP-3202 (“Sensation and Perception”) during the Fall 2021 semester. All participants were given the opportunity to complete three experiments (**QT-1**, **QT-2**, **QT-3**) implemented as web surveys in return for course credit.

#### Procedures

The visual experiments were implemented as a web survey using Qualtrics, an online web survey program (https://www.qualtrics.com/). Participants accessed the link for the Qualtrics survey using their personal computers or smartphones. Image patches used in the Qualtrics surveys used in this study were selected from the McGill Calibrated Color Image Database [9] and then converted to grayscale. In each survey, participants were shown a 40x40 image patch (resized to 96x96, in order to subtend roughly 2–3 degrees of visual angle at normal viewing distances) and asked to indicate whether this patch contained an occlusion edge or a shadow edge. Each survey contained 200 images, half of which belonged to each category, presented in random order. After their response, feedback (correct/incorrect) was provided before presenting the next stimulus. A schematic diagram of the experimental procedure is shown in **Fig 10A**, and an example survey question is shown in**. S5 Fig**.

For inclusion in the final analysis, students had to complete all three surveys in the correct order, have no blank responses, and perform better than chance (114 or more correct out of 200) on the final 2 surveys, indicating that they had obtained proficiency in the task. Although there were 37 participants who completed at least one survey, only 18 participants met the criteria for inclusion in the final analysis, mostly since many participants failed to complete all three surveys.

### Psychophysical experiment: Laboratory

#### Participants

Participants were undergraduate students attending Florida Gulf Coast University in Spring of 2022 who were enrolled in Psychology courses PSY-3205 (“Survey of Analytical Techniques”) and EXP-3202 (“Sensation and Perception”). As before, participants were required to review and accept an informed consent form prior to the experiment and were compensated with course credit. All procedures were approved beforehand by the FGCU IRB (Protocol 2014–01).

#### Procedures

Observers performed the exact same single interval forced choice task as the Qualtrics surveys using the exact same stimulus sets, with randomized presentation order, and auditory feedback after each response. Stimuli were displayed on a calibrated, gamma-corrected (gamma = 1.0) Display++ monitor (Cambridge Research) with a mid-point luminance of 100 cd/m^2^. All images were set to 12.5% RMS contrast and scaled to subtend 2 degrees of visual angle at the viewing distance of 52 cm. We refer to these laboratory (**LB**) versions of the Qualtrics surveys as **LB-1**, **LB-2**, **LB-3**. For inclusion in the final analysis, participants had to complete all three surveys, with better-than-chance performance on each of the final two. 17 participants completed all surveys, and of these 15 satisfied the criteria for inclusion.

## Supporting information

S1 Table Logistic regression classifier analysis for oset-1. Each block used the indicated images as the test set and trained on the remainder. This ensured no overlap between the test and training sets. The 2D in parentheses refers to classification using only Michelson and RMS Contrast parameters. The 3D analyses also include a parameter measuring proportion of stimulus energy in high spatial frequencies. **occ** = occlusion images, **shad** = shadow images.
(XLSX)

S2 Table Same as S1 Table but for occlusions taken from oset-2. (XLSX)

S3 Table Gabor Filter Bank (GFB) classifier analysis for oset-1 at the best hyper-parameter value. Each block used the indicated images as the test set and trained on the remainder.
(XLSX)

S4 Table Same as S3 Table, but for oset-2. (XLSX)

S5 Table Classifier performance on 4 test sets not used for training models. In this analysis, both the training sets and the test sets were sampled uniformly with replacement from all the images.
(XLSX)

S6 Table Filter-Rectify-Filter model with 6 hidden units (FRF-6) classifier analysis for oset-1. Each block used the indicated images as the test set and trained on the remainder.
(XLSX)

S7 Table Same as S6 Table, but for oset-2. (XLSX)

S8 Table Filter-Rectify-Filter model with 4 hidden units (FRF-4) classifier analysis for oset-1. (XLSX)

S9 Table Same as S8 Table, but for oset-2. (XLSX)

S10 Table Filter-Rectify-Filter model with 10 hidden units (FRF-10) classifier analysis for oset-1. (XLSX)

S11 Table Same as S10 Table, but for oset-2. (XLSX)

S1 Fig Examples of three images from oset-1 [3] which contain surface boundaries which are not depth occlusions, but rather two different surfaces juxtaposed in the same plane. (TIF)

S2 Fig Same as Fig 2A in main text but for a subset of 19 images in oset-1 which were analyzed by [7]. (TIF)

S3 Fig Same as Fig 2A in main text but for image patches which were normalized to the range [0, 1]. (TIF)

S4 Fig log-Gabor filters at one spatial scale (16x16) used in both the GFB model and FRF models (Fig 4). (TIF)

S5 Fig Example question from the Qualtrics (QT) surveys. (TIF)

S6 Fig Same as Fig 11 in the main text for the LB surveys. (TIF)

S7 Fig Scatterplots of the occlusion probability from the AO model (horizontal axis) and the GFB, FRF models (vertical axis) for the QT surveys. Magenta dots indicate shadows, green indicates occlusions.
(TIF)

S8 Fig Same as Fig 12 in the main text for the LB surveys. Magenta dots indicate shadows, green indicates occlusions.
(TIF)

S9 Fig Analysis of classification bias in individual human observers. (**a**) Distribution of the difference *P*(*o*|*S*)−*P*(*s*|*O*) in classification error probabilities for **QT** surveys. *P*(*o*|*S*) indicates the probability a shadow is misclassified as an occlusion, and *P*(*s*|*O*) is the probability of an occlusion being misclassified as a shadow (N = 54). (**b**) Distribution of the Wald statistic for the binomial proportion test testing whether there is a bias in misclassifications, with the null hypothesis of equal misclassification probabilities. (**c**) Scatter plot of classification error probabilities. (**d**) Same as (a) but for **LB** surveys (N = 45) (**e**) Same as (b) but for **LB** surveys. (**f**) Same as (c) but for **LB** surveys.
(TIF)

S10 Fig Decision-variable correlation analysis between models and individual observers for each experiment. *Left*: **QT** surveys. *Center*: **LB** surveys. *Right*: Pooled **QT** + **LB** surveys.
(TIF)

S1 Code Python code for the FRF and GFB models described in the text. (ZIP)

S1 Text Supplementary Results. (DOCX)
