# Supplementary figures and images for: Distinguishing shadows from surface boundaries using local achromatic cues
Source: PLoS Comput Biol. 2022 Sep 14;18(9):e1010473. doi: 10.1371/journal.pcbi.1010473 (PMC9512248; doi:10.1371/journal.pcbi.1010473)

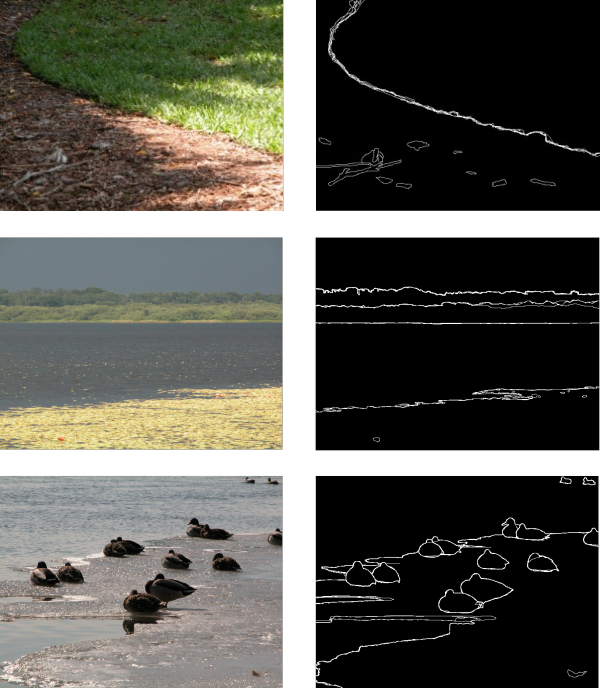

Supplement: S1 Fig — (TIF) [file pcbi.1010473.s012.tif]

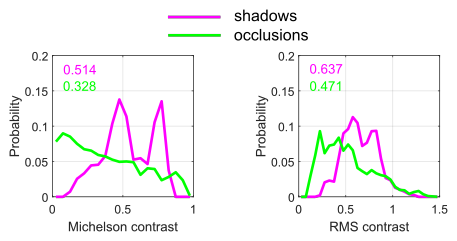

Supplement: S2 Fig — (TIF) [file pcbi.1010473.s013.tif]

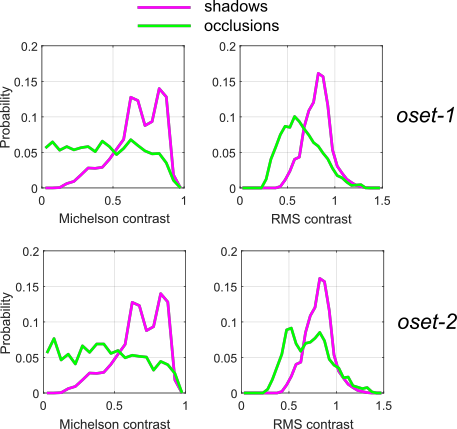

Supplement: S3 Fig — (TIF) [file pcbi.1010473.s014.tif]

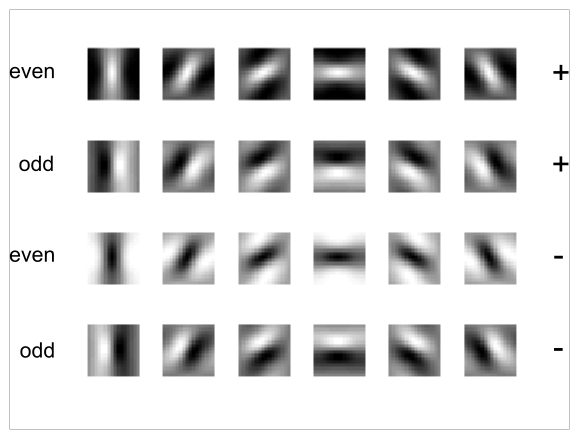

Supplement: S4 Fig — (TIF) [file pcbi.1010473.s015.tif]

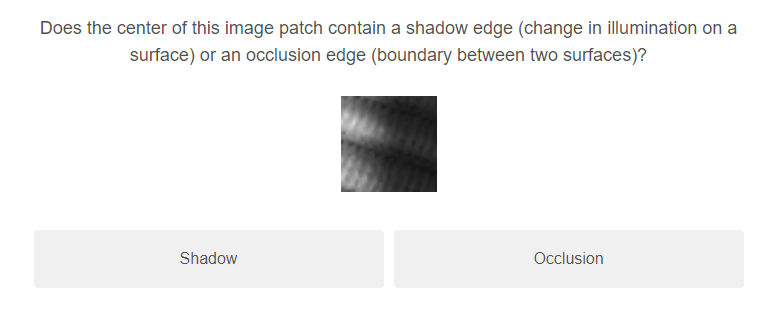

Supplement: S5 Fig — (TIF) [file pcbi.1010473.s016.tif]

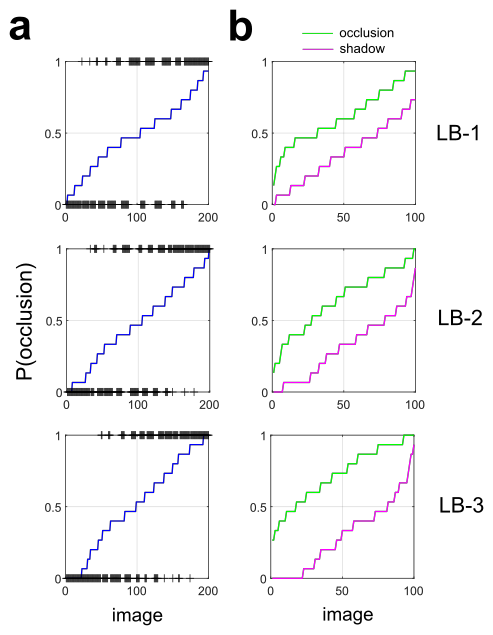

Supplement: S6 Fig — (TIF) [file pcbi.1010473.s017.tif]

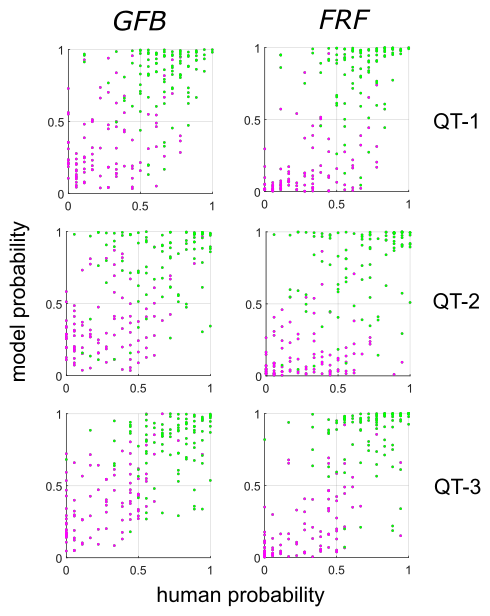

Supplement: S7 Fig — Magenta dots indicate shadows, green indicates occlusions. (TIF) [file pcbi.1010473.s018.tif]

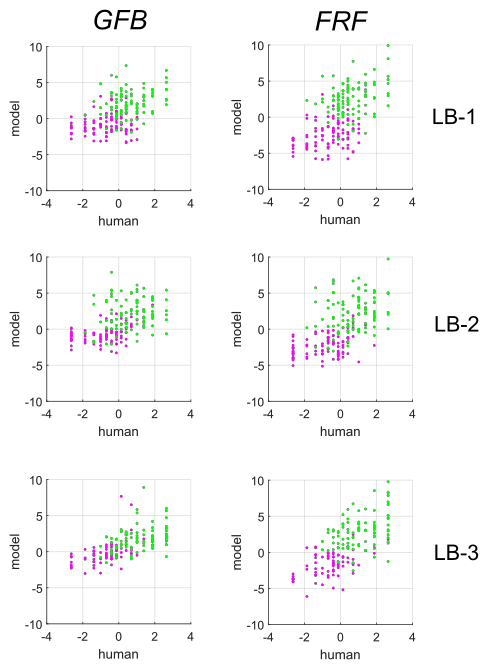

Supplement: S8 Fig — Magenta dots indicate shadows, green indicates occlusions. (TIF) [file pcbi.1010473.s019.tif]

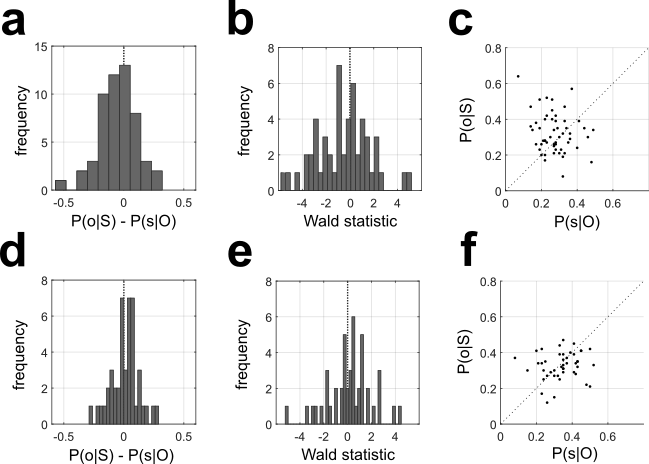

Supplement: S9 Fig — (a) Distribution of the difference P(o|S)−P(s|O) in classification error probabilities for QT surveys. P(o|S) indicates the probability a shadow is misclassified as an occlusion, and P(s|O) is the probability of an occlusion being misclassified as a shadow (N = 54). (b) Distribution of the Wald statistic for the binomial proportion test testing whether there is a bias in misclassifications, with the null hypothesis of equal misclassification probabilities. (c) Scatter plot of classification error probabilities. (d) Same as (a) but for LB surveys (N = 45) (e) Same as (b) but for LB surveys. (f) Same as (c) but for LB surveys. (TIF) [file pcbi.1010473.s020.tif]

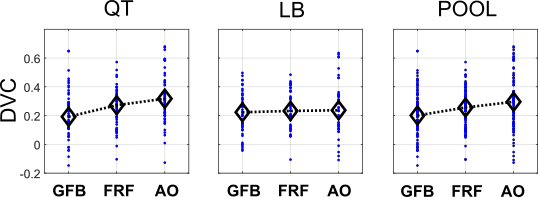

Supplement: S10 Fig — Left: QT surveys. Center: LB surveys. Right: Pooled QT + LB surveys. (TIF) [file pcbi.1010473.s021.tif]
