# Supplementary material for: Distinguishing shadows from surface boundaries using local achromatic cues
Source: PLoS Comput Biol. 2022 Sep 14;18(9):e1010473. doi: 10.1371/journal.pcbi.1010473 (PMC9512248; doi:10.1371/journal.pcbi.1010473)
Supplement: S1 Text — (DOCX) [file pcbi.1010473.s023.docx]

**SUPPLEMENTARY RESULTS**

Analysis of misclassification by individuals

Since one of our goals is to compare human performance to that of machine observers, we want to not only compare the probability of correct response, but also the pattern of classification errors. For each observer and experiment, we measured the probability $P\left( s | O \right)$ that an occlusion was misclassified as a shadow, and the probability $P\left( o | S \right)$ that a shadow was misclassified as an occlusion. If there is no bias in either direction, the difference $P\left( s | O \right)-P\left( o | S \right)$(**S9a Fig**) should be zero. A positive value would indicate a greater likelihood of misclassifying occlusions as shadows than misclassifying shadows as occlusions. A negative value would indicate a greater likelihood of misclassifying shadows as occlusions than the other way around. On an individual basis, this can be assessed using a difference of binomial proportion test (*Wald test*). Doing so for the **QT** surveys revealed that over 54 observer-surveys, in 22/54 cases a significant bias was observed towards misclassifying one or the other stimulus categories. Of these, 6/22 observer-surveys exhibited a bias to misclassify occlusions as shadows, whereas in 16/22 observer-surveys we saw a bias to misclassify shadows as occlusions. The proportion of observer-surveys which exhibited a significant bias to misclassify shadows as occlusions does not differ significantly from chance (binomial proportion test, N = 22, *p* = 0.055). Similar results were found for the **LB** surveys (**S9d Fig**). For 11/45 observer-surveys we observed a bias, with 5/11 exhibiting a bias towards edges and 6/11 a bias towards shadows (not significantly different).

Over the full set of **QT** observer-surveys, comparing the median Wald statistic (**S9b Fig**) to zero (sign-rank test) failed to reach the conventional criterion for statistical significance (median = -0.675, *p* = 0.057, N = 54). Over the full set of **LB** surveys, the Wald statistic (**S9e Fig**) does not differ significantly from zero (sign-rank test, median = 0.301, *p* = 0.296, N = 45). **S9c Fig** shows a scatterplot of $P\left( s | O \right)$ and $P\left( o | S \right)$ for the **QT** surveys, and we see that points are about equally likely to be above and below the diagonal, with similar findings for the **LB** surveys (**S9f Fig**).

Analysis of misclassification patterns by the AO model

Another way to compare the performance of the models to human observers is to examine the patterns of classification errors. If there is no bias in either direction in the pattern of misclassification errors, then the proportion of misclassifications of shadows as occlusions (or vice versa) should not differ from 0.5. This can be assessed for each machine classifier (**GFB**, **FRF**) and the **AO** (**Tables 1**, **2**) using a simple binomial proportion test. Here we list the proportion of classification errors which involved classifying the patch as an occlusion, together with the 95% CI of the null hypothesis of equal probabilities of errors in either direction. Pooling across all surveys, for the **GFB**, 35/111 misclassifications are in the direction of occlusions, with *p* < 0.001 (binomial proportion test, N = 111). By contrast, for the **FRF** model 32/56 misclassifications are in the direction of occlusions, and we fail to see a significant bias (*p* = 0.3496, N = 56). For the **AO** derived from the **QT** surveys, 50/114 misclassifications favor occlusions, exhibiting no significant bias (*p* = 0.2234, N = 114). For the **AO** derived by pooling across the **LB** surveys, 68/143 misclassifications favor occlusions, also exhibiting no bias (*p* = 0.616, N = 143). Thus, we see a much stronger agreement between the **FRF** model and average human behavior (**AO**) based on patterns of misclassification.

Decision variable correlation

We further explored the relative efficacy of the **FRF** and **GFB** models in accounting for human behavior using Decision Variable Correlation (**DVC**), an extension of signal detection theory [1] which has been broadly applied in various psychophysical domains [2, 3, 4, 5]. This novel methodology assumes that for classification tasks both a human observer and a model (or two humans) make use of an internal, unobserved decision variable which each compares to some criterion to decide which stimulus was presented. Assuming the joint distribution of the human and model decision variables is bivariate normal, by analyzing the patterns of agreement and disagreement, it is possible to recover the covariance. DVC is particularly powerful for model selection, since it is possible for two models to predict performance (as percent correct) equally well, while not agreeing on which particular stimuli the human and model classify into each category.

**S10 Fig** (*left*) shows DVCs computed from all the **QT** surveys. We observe significantly better performance (Wilcoxon related-samples signed-rank test) for the **FRF** model than the **GFB** model (median **FRF**-**GFB** = 0.045, *p* = 0.005, N = 54), although we see the best performance for the **AO** model (median **AO-GFB** = 0.142, *p* = 0.001; median **AO**-**FRF** = 0.061, *p* = 0.021), where the **AO** is derived from all of the other observers. **S10 Fig** (*middle*) shows the DVC computed from all of the **LB** surveys. As with the **QT** surveys, for the **LB** surveys we observe significantly better performance for the **FRF** model than the **GFB** model (median **FRF**-**GFB** = 0.0206, *p* = 0.03, N = 45). However, for the **LB** surveys due to variability in the DVC computed from the **AO**, we do not observe significant differences between the **AO** and either of the machine classifiers (median **AO-GFB** = 0.026, *p* = 0.229; median **AO**-**FRF** = 0.018, *p* = 0.584, N = 45). Pooling results from the two experiments (**S10 Fig**, *right*) shows better performance for the **AO** than either machine classifier (median **AO-GFB** = 0.086, *p* < 0.001, N = 99; median **AO-FRF** = 0.033, *p* = 0.037), and better performance by the **FRF** model than the **GFB** model (median **FRF-GFB** = 0.040, *p* < 0.001). This is convergent with our earlier analyses suggesting that the **FRF** model is in better agreement with the human observers than the **GFB** model.

**SUPPLEMENTARY REFERENCES**

[1] Sebastian, S. & Geisler, W.S. (2018). Decision-variable correlation. *Journal of Vision*, *18*(4), 3-3.

[2] DiMattina, C., & Baker, C.L., Jr. (2019). Modeling second-order boundary perception: A machine learning approach. *PLoS Computational Biology*, *15*(3), e1006829.

[3] Chin, B. M., & Burge, J. (2020). Predicting the partition of behavioral variability in speed perception with naturalistic stimuli. *Journal of Neuroscience*, *40*(4), 864-879.

[4] Henry, K. S., & Abrams, K. S. (2021). Normal tone-in-noise sensitivity in trained budgerigars despite substantial auditory-nerve injury: no evidence of hidden hearing loss. *Journal of Neuroscience*, *41*(1), 118-129.

[5] Sebastian, S., Seemiller, E. S., & Geisler, W. S. (2020). Local reliability weighting explains identification of partially masked objects in natural images. *Proceedings of the National Academy of Sciences*, *117*(47), 29363-29370.
